# Lung tumorigenesis induced by human vascular endothelial growth factor (hVEGF)-A_165_ overexpression in transgenic mice and amelioration of tumor formation by miR-16

**DOI:** 10.18632/oncotarget.3390

**Published:** 2015-04-22

**Authors:** Yu-Tang Tung, Pin-Wu Huang, Yu-Ching Chou, Cheng-Wei Lai, Hsiu-Po Wang, Heng-Chien Ho, Chih-Ching Yen, Chih-Yen Tu, Tung-Chou Tsai, Dah-Cherng Yeh, Jiun-Long Wang, Kowit-Yu Chong, Chuan-Mu Chen

**Affiliations:** ^1^ Department of Life Sciences, and Agricultural Biotechnology Center, National Chung Hsing University, Taichung 402, Taiwan; ^2^ Department of Medicine, China Medical University Hospital, Taichung 404, Taiwan; ^3^ Department of Internal Medicine, China Medical University Hospital, Taichung 404, Taiwan; ^4^ Department of General Surgery and Department of Internal Medicine, Taichung Veterans General Hospital, Taichung 407, Taiwan; ^5^ Division of Chest Medicine, Department of Internal Medicine, Taichung Veterans General Hospital, Taichung 407, Taiwan; ^6^ Department of Medical Biotechnology and Laboratory Sciences, College of Medicine, Chang Gung University, Tao-Yuan 333, Taiwan; ^7^ Molecular Medicine Research Center, College of Medicine, Chang Gung University, Tao-Yuan 333, Taiwan; ^8^ Rong-Hsing Translational Medicine Center and iEGG Center, National Chung Hsing University, Taichung 402, Taiwan

**Keywords:** VEGF, transgenic mice, pulmonary tumorigenesis, magnetic resonance imaging (MRI), miRNA therapy

## Abstract

Many studies have shown that vascular endothelial growth factor (VEGF), especially the human VEGF-A_165_ (hVEGF-A_165_) isoform, is a key proangiogenic factor that is overexpressed in lung cancer. We generated transgenic mice that overexpresses hVEGF-A_165_ in lung-specific Clara cells to investigate the development of pulmonary adenocarcinoma. In this study, three transgenic mouse strains were produced by pronuclear microinjection, and Southern blot analysis indicated similar patterns of the foreign gene within the genomes of the transgenic founder mice and their offspring. Accordingly, *hVegf-A_165_* mRNA was expressed specifically in the lung tissue of the transgenic mice. Histopathological examination of the lung tissues of the transgenic mice showed that hVEGF-A_165_ overexpression induced bronchial inflammation, fibrosis, cysts, and adenoma. Pathological section and magnetic resonance imaging (MRI) analyses demonstrated a positive correlation between the development of pulmonary cancer and hVEGF expression levels, which were determined by immunohistochemistry, qRT-PCR, and western blot analyses. Gene expression profiling by cDNA microarray revealed a set of up-regulated genes (*hvegf-A_165_*, *cyclin b1*, *cdc2*, *egfr*, *mmp9*, *nrp-1*, and *kdr*) in VEGF tumors compared with wild-type lung tissues. In addition, overexpressing hVEGF-A_165_ in Clara cells increases CD105, fibrogenic genes (*collagen α1*, *α-SMA*, *TGF-β1*, and *TIMP1*), and inflammatory cytokines (IL-1, IL-6, and TNF-*α*) in the lungs of hVEGF-A_165_-overexpressing transgenic mice as compared to wild-type mice. We further demonstrated that the intranasal administration of microRNA-16 (miR-16) inhibited lung tumor growth by suppressing VEGF expression *via* the intrinsic and extrinsic apoptotic pathways. In conclusion, hVEGF-A_165_ transgenic mice exhibited complex alterations in gene expression and tumorigenesis and may be a relevant model for studying VEGF-targeted therapies in lung adenocarcinoma.

## INTRODUCTION

Despite major advances in cancer therapeutics, the survival rate for patients with lung cancer has not significantly improved, even in the era of targeted therapy [1]. Lung cancer is responsible for 1.3 million annual deaths worldwide [2], and it remains the leading cause of cancer-related deaths in the western world, with approximately 160,000 deaths annually [3]. It is clear that chemotherapy effectiveness has reached a plateau [4], and newer, more active approaches are needed to improve the prognosis of this devastating disease. Thus, mouse models for human lung cancer have been developed in recent years to understand the mechanisms of the disease, to identify several potential molecular targets and to develop novel “targeted therapies”.

The primary cause of pulmonary cancer consists of gene mutations induced by carcinogens contained in items, such as the cigarette smoke inhaled by long-term addicts or environmentally polluted air. The number of the gene mutations that accumulate over time may promote cell carcinogenesis and the growth of a tumor. In addition to cigarette addiction, the long-term inflammatory response in the lungs caused by allergens in the air may also cause pulmonary adenocarcinoma. Thus, chronic diseases in the lungs and family inheritance are two common causes of pulmonary adenocarcinoma. According to previous studies, genetic background is another cause of pulmonary cancers. A person with a family history of pulmonary cancer will have a higher probability of pulmonary carcinogenesis [5]. Additionally, there is evidence that gene mutations (*k-ras*, *cyclin d1*, *bcl2*, *rb*, and *apc*) play a role in the formation of human lung adenocarcinoma [6, 7].

Angiogenesis is one of the crucial causes of cancer development, and vascular endothelial growth factor (VEGF) overexpression is associated with poor prognosis in patients with non-small cell lung cancer (NSCLC). Therefore, angiogenesis-targeting agents were among the first to be recognized for their potential benefit against NSCLC [8]. Several proangiogenic factors have been identified, and VEGF is considered the most potent [9]. The abnormal function of VEGF results in many diseases, such as cardiovascular diseases, pulmonary edema, improper inflammation response, tumor metastasis, and angiogenesis [10]. The VEGF gene family consists of four homologues: VEGF-A (usually referred to as VEGF), VEGF-B, VEGF-C, and VEGF-D. VEGF-A is considered the most important for both physiological and pathological angiogenesis [9, 11]. Additionally, under normal regulatory conditions, VEGF-A helps in wound occlusion and in regulating the female menstrual cycle [12]. A cell or a tissue under hypoxia or ischemia is able to induce the expression of the hypoxia-inducible factor-1 (HIF-1) protein to promote the efficiency of *VEGF-A* mRNA transcription, to promote the occurrence of the VEGF-A protein, and to cause angiogenesis. Moreover, VEGF-A is divided into four isoforms, including VEGF-A_121_, VEGF-A_165_, VEGF-A_189_, and VEGF-A_206_. VEGF-A_165_, the most common form of VEGF-A, primarily functions in promoting angiogenesis. When VEGF-A lacks exon 6, the resulting protein, VEGF-A_165_, still retains the heparin-binding site, but its ability to link to acetyl heparin sulfate is much lower than that of the two isoforms VEGF-A_206_ and VEGF-A_189_.

Early-stage cancer cells will continue to proliferate, leading to nutrition and oxygen deficiencies and resulting in a large amount of cell death. Therefore, an inflammatory response will occur, and HIF-1α will be activated to induce the secretion of a large quantity of VEGF-A_165_. VEGF-A_165_ will then bind to VEGFR2, activating downstream signals to induce vasculogenesis [12, 13]. It is evident that VEGF-A_165_ supports the growth and metastasis of malignant tumor cells. When cancer cells secrete a large amount of VEGF-A_165_, vasculogenesis is induced to provide a sufficient amount of nutrients and oxygen to the tumor, thus increasing the rate of tumor growth [14]. The over-secretion of VEGF-A_165_ promotes degradation of the extra-cellular matrix and increases vascular permeability, rendering the tumor cells liable to invade other tissues [15]. Therefore, the development of inhibitors and therapies targeting VEGF-A_165_ and its related factors are popular areas of research. Here, we developed a method for generating transgenic mice that express VEGF-A_165_; these animals can serve as a model for investigating the regulatory mechanism of pulmonary adenocarcinoma.

## RESULTS

### Production of transgenic mice carrying the *mccsp-Vegf-A_165_*-sv40** transgenic fusion gene

**Figure 1:**
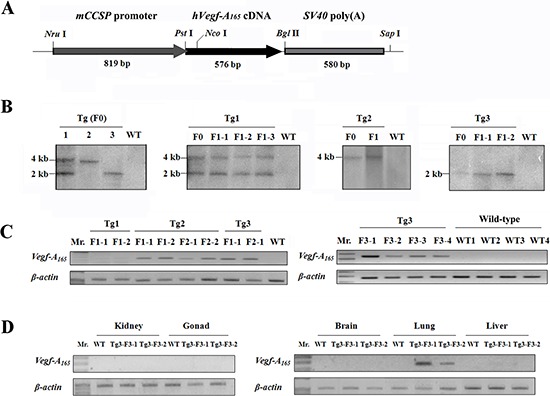
Schematic map of the hVEGF-A_165_ transgene construct and detection of its integration into the transgenic mouse genome **A.** hVEGF-A_165_ overexpression construct controlled by mouse Clara cell-specific protein (mccsp) promoter. **B.** Verification of germline transmission and the transgenic patterns in the genomic DNA of the transgenic mice by Southern blot analysis. The Southern blot data showed that the foreign gene was found in similar patterns within the genomes of the transgenic founder mice and their offspring. **C.** The pulmonary *Vegf-A_165_* mRNA expression level in the offspring of transgenic mice was determined by semi-quantitative RT-PCR. The data showed that the *Vegf-A_165_* mRNA was expressed in the Tg 2 and Tg 3 offspring, but it was not expressed in the Tg 1 offspring. β-actin was used as an internal control. **D.** The *Vegf-A_165_* mRNA expression in specific tissues was determined using semi-quantitative RT-PCR. RT-PCR showed that *Vegf-A_165_* mRNA was expressed specifically in the lung tissue of transgenic mice. β-actin was used as an internal control.

A 1,975-bp *mccsp-Vegf-A_165_-SV40* transgene (Figure 1A) was directly microinjected into pronuclear-stage mouse embryos, which were then transferred into the fallopian tubes of recipient females. After parturition, 36 newborn mice were obtained and underwent PCR screening, and 3 newborn mice, named Tg1-F0, Tg2-F0, and Tg3-F0, were identified to have successful integration of the transgene. Each of the three transgenic mice was mated to a normal FVB mouse to produce offspring (F1, F2 and F3). The integration pattern of the foreign gene was determined by Southern blot analysis (Figure 1B), and three transgenic founders were found to possess different transgenic patterns. Multiple integrated copies of the transgene were found to be stably inserted into the germ lines of these founders. To determine the expression level of the transgene in the transgenic mice, semi-quantitative reverse transcription-PCR (RT-PCR) was performed. As shown in Figure 1C, a 243-bp RT-PCR product was amplified from the lung tissue of transgenic mice. The transgenic line Tg3 exhibited a higher level of *hvegf-A_165_* expression than the transgenic line Tg2, while no *hvegf-A_165_* expression was detected in the transgenic line Tg1 or the wild-type mice. Therefore, the transgenic founders of the transgenic line Tg3 had the highest *hvegf-A_165_* expression. Additionally, the *hvegf-A_165_* mRNA of the transgenic line Tg3-F3-1 was specifically expressed in the lungs of the mouse and was not expressed in the tissues of other organs, including the kidney, gonad, brain, and liver (Figure 1D).

### Histology of the transgenic mice

**Figure 2:**
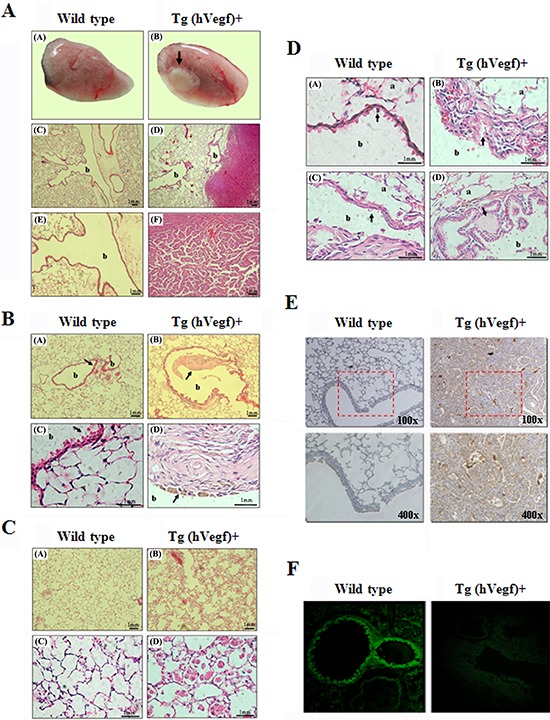
The exterior and histopathologic staining and immunohistochemical (IHC) staining of lung tissues from wild-type and transgenic mice **A.** Exterior and histopathologic section images of the lung tissues in the mice: pulmonary adenomas. (A) and (B) present the exterior of the lungs from wild-type and transgenic mice, respectively. The arrows in (B) indicate adenomas. (C) The bronchia and alveoli of lung tissue from a wild-type mouse are shown. (D) The bronchia, alveoli and adenomas of lung tissue from a transgenic mouse are shown. (E) and (F) show the 2.5X magnification images of (C) and (D), respectively. (“b” indicates the bronchia). **B.** Histopathologic section images of the lung tissues from the mice: fibrosis. (A) The bronchia and alveoli of the lung tissue in a wild-type mouse are shown. (B) The fibrosis (arrow) in the bronchia of the transgenic mice was due to bleeding. (C) and (D) are the 6X magnification images of (A) and (B), respectively (“b” indicates the bronchia). **C.** Histopathologic section images of lung tissues from the mice: inflammation. (A) The normal alveoli in a wild-type mouse are shown. (B) Inflammation of the alveoli in a transgenic mouse is shown. (C) and (D) are the 6X magnification images of (A) and (B), respectively. **D.** Histopathologic section images of lung tissues from the mice: abnormal bronchial epithelium. (A) The normal bronchia in a wild-type mouse are shown. (B) The proliferation of the cells (arrow) on the bronchial epithelium of a transgenic mouse is shown. (C) The flattened bronchial epithelium (arrow) of a transgenic mouse is shown. (D) The cyst (arrow) on the bronchial epithelium of a transgenic mouse is shown. (“b” indicates the bronchia, “a” indicates the alveoli). **E.** Detection of hVEGF-A_165_ expression in the lung tissue of mice by IHC staining is shown. **F.** Detection of the bronchia mCCSP in the lung tissue of mice using fluorescence microscopy is shown.

We performed serial histologic analysis of the lungs of transgenic and wild-type mice up to the age of 12 months. The wild-type mice showed normal lung histology with no evidence of malignancy (Figure 2A, left). In contrast, the transgenic mice began to develop neoplasms on the periphery of the pulmonary alveolus and adenomas near the lung bronchus (Figure 2A, right). In addition, we also observed bronchial fibrosis in the transgenic mice (Figure 2B, right) but normal bronchial walls in the wild-type mice (Figure 2B, left). Compared with the wild-type mice, the transgenic mice had an increased number of intra-alveolar macrophages with strong eosinophilic cytoplasm, which presumably represented an obvious inflammation response (Figure 2C, right). A cyst created by hyperplasia of the epidermis on the bronchus and blockage of the secretory tissues further induced pulmonary emphysema in the transgenic mice (Figure 2D, right). Additionally, both local cell hyperplasia and flattening phenomena on the epidermis of the bronchus of the transgenic mouse were also found (Figure 2D, right). According to the results, the inflammatory response in the bronchus to alveoli may have been induced by overexpression of hVEGF-A_165_. Furthermore, immunoblot analysis confirmed that an increased hVEGF-A_165_ protein level was detected in the lung tissue of transgenic mice (Figure 2E, right) and that no hVEGF-A_165_ protein was found in the wild-type mice (Figure 2E, left). Therefore, exogenous hVEGF-A_165_ protein was specifically expressed in the Clara lung cells of the transgenic mice, and the expression level of this protein was noticeably higher in the transgenic mice than the wild-type mice. As shown in Figure 2F, the expression of fluorescently labeled CCSP was found in the bead-like Clara cells in lung capillary bronchus sections from the wild-type mice (Figure 2F, left), while only very weak expression of the fluorescently labeled CCSP was found in the transgenic mice (Figure 2F, right).

### hVEGF-A_165_ expression in three tumorigenesis levels of transgenic mice

**Figure 3:**
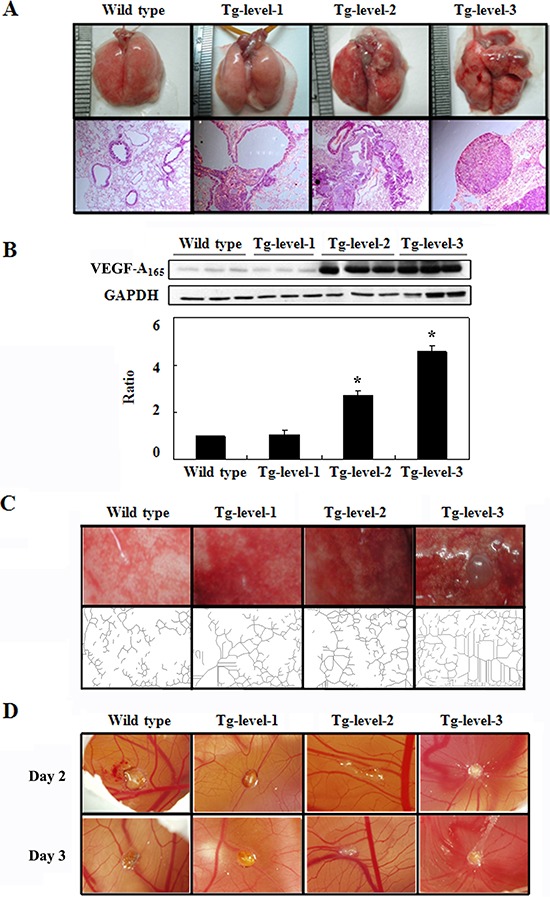
The exterior and histopathologic sections and neovascular and angiogenic activity of the lung tissues in the wild-type mice and in the three tumorigenesis levels of transgenic mice (Tg-level-1, Tg-level-2, and Tg-level-3) **A.** Exterior and histopathologic section images of the lung tissues of the mice. **B.** hVEGF-A_165_ protein expression in the lung tissues of mice was determined by western blot analysis; mean ± SD (*n* = 3). **C.** The neovasculature of the lung tissues of the mice was analyzed using an Angiogenesis Image Analyzer. **D.** The angiogenic activity of the lung tissues of the mice was analyzed using chick chorioallantoic membrane (CAM).

To analyze the relative expression levels of hVEGF-A_165_, we divided the > 12-month-old transgenic mice into three groups, Tg-level-1, Tg-level-2, and Tg-level-3 (Figure 3A). The Tg-level-1 transgenic mice showed no obvious differences in their exterior lung tissues. However, the lung tissues of the Tg-level-2 transgenic mice exhibited red color block distribution in their exterior lung tissues. The lung tissues of the Tg-level-3 transgenic mice had serious injury to their exterior lung tissues and had formed a tumor protrusion. As shown in Figure 3B, the transgenic mice of the three tumorigenesis levels showed differential hVEGF-A_16*5*_ protein expression by western blot analysis. The hVEGF-A_16*5*_ protein expression level was significantly higher by 2.7- and 4.5-fold in the Tg-level-2 and Tg-level-3 transgenic mice, respectively, compared with that of the Tg-level-1 as well as wild type mice (*p* < 0.05). By matching the hVEGF-A_165_ protein expression level to the lung tissues of the three tumorigenesis levels of transgenic mice, it was speculated that both the injuries and carcinogenesis of the transgenic mice were positively related to a specific hVEGF-A_165_ protein expression level in the lung.

Additionally, the quantitation of the angiogenesis of lung-tissue spreads using an Angiogenesis Image Analyzer (Kurabo, USA) is shown in Figure 3C. The number of branches of angiogenesis was significantly higher by 2.0-, 2.7-, and 3.9-fold in Tg-level-1, Tg-level-2, and Tg-level-3 transgenic mice, respectively, compared with that of the wild type mice (*p* < 0.05). To evaluate the angiogenic activity of the three tumorigenesis levels of the transgenic mice, chick embryo chorioallantoic membranes (CAMs) were used, and the results are shown in Figure 3D. There was obvious hyperplasia in the lung tissues of the Tg-level-2 and Tg-level-3 transgenic mice that clearly produced a larger vascular zone in CAMs compared with the lung tissue of wild-type mice (Figure 3D). However, there were no significant differences between the lung tissues of wild-type mice and Tg-level-1 transgenic mice.

### The cDNA microarray analysis

**Figure 4:**
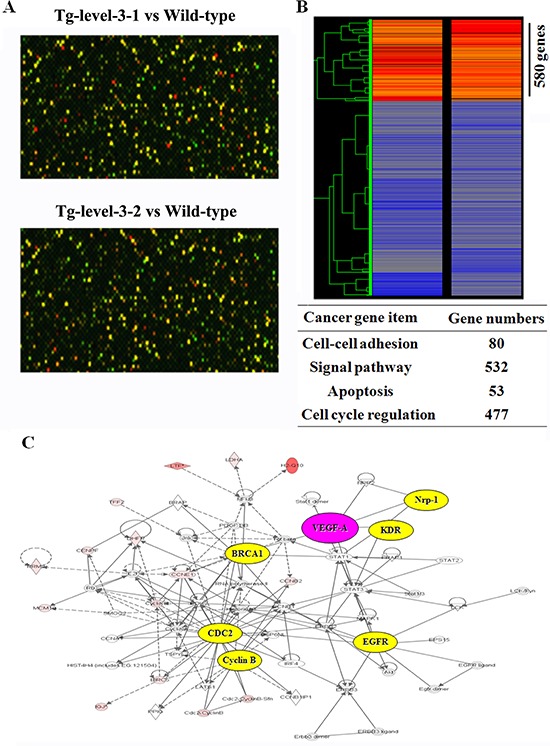
The cDNA microarray data **A.** Representative images of the lung tissues of wild-type and transgenic mice (Tg-level-3/wild type) after using two-color fluorescent probe hybridization. **B.** Clustar display of a mouse cDNA microarray of the lung tissues of wild-type and transgenic mice (Tg-level-3/wild type). The Cy5/Cy3 ratios of 580 cDNAs that deviated by more than 2-fold are shown. **C.** The ingenuity pathway analysis (IPA) of the mouse cDNA microarray data obtained from wild-type and Tg-level-3 mice.

The differences in the gene expression profiles of the transgenic and wild-type mice were investigated using cDNA microarrays to understand adenocarcinomagenesis, and the results are as shown in Figure 4. Using an Agilent mouse whole genome 44K oligonucleotide microarray, the lung tissue expression patterns of 44,000 known genes were analyzed. Of the 3,000 genes represented on the microarray, we selected genes that were at least 2-fold differentially expressed, and of these genes, 580 (cell-cell adhesion: 80, signal pathway: 532, apoptosis: 53, and cell cycle regulation: 477) were up-regulated in the lung tissue of Tg-level-3 compared with wild-type mice, as shown in Supplementary Table 1. Furthermore, the microarray data were analyzed using Ingenuity Pathway Analysis (IPA) software. The IPA result showed that a cluster of genes were triggered by overexpressed hVEGF-A_165_, and these genes are targets of cancer development, respiratory disease, and cell cycle regulators.

**Figure 5:**
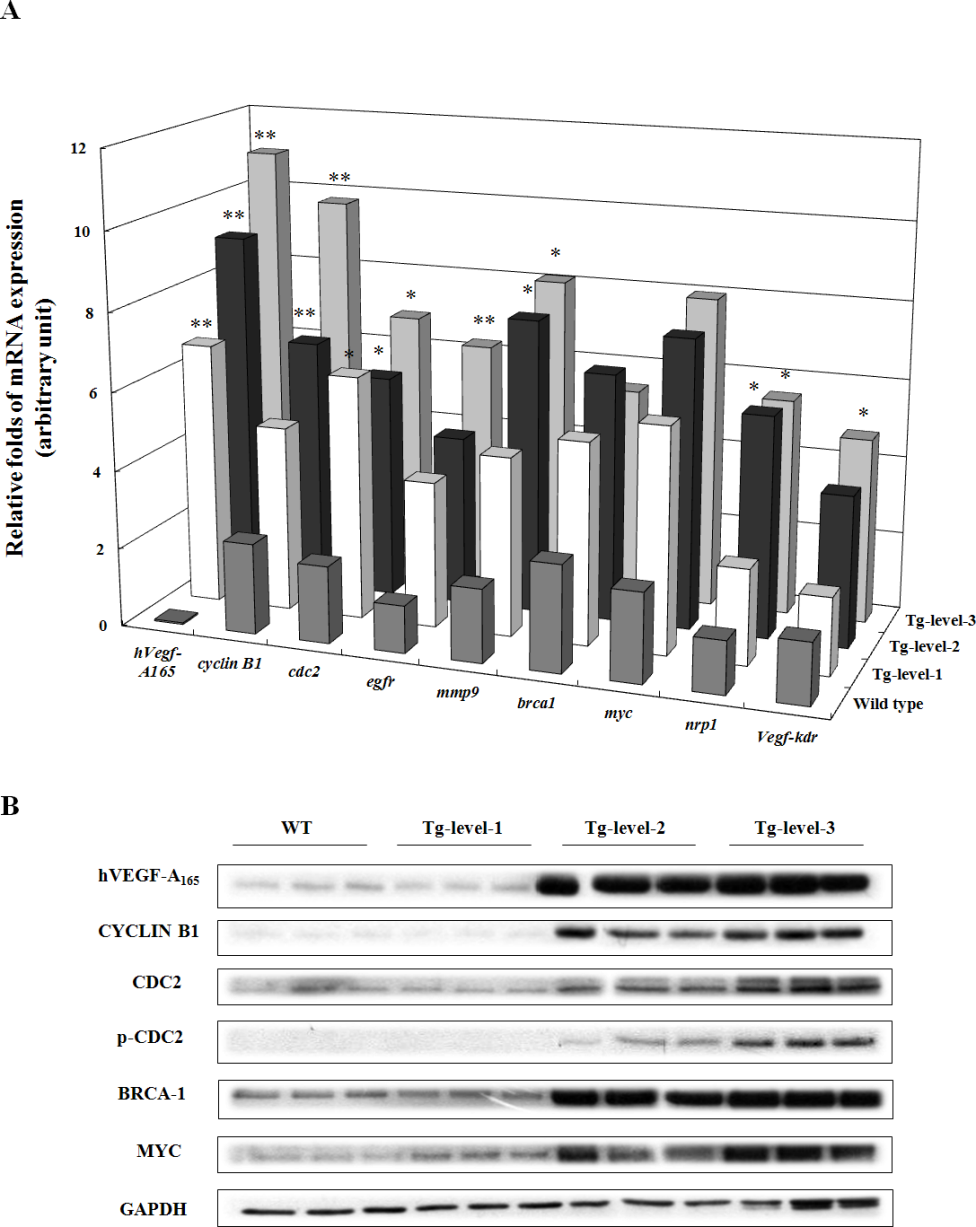
The expression levels of mRNA and protein in the lung tissues of wild-type mice and of three different tumorigenesis levels of transgenic mice (Tg-level-1, Tg-level-2, and Tg-level-3) **A.** The quantitative mRNA expression levels of the *hVegf-A_165_*, *cyclin b1* (cell cycle), *cdc2* (cell cycle), *egfr* (cell proliferation), *mmp9* (cell migration), *brca1* (oncogene), *myc* (oncogene), *nrp1* (co-receptor), and *vegfr2-kdr* (receptor) genes in the lung tissues of the mice were determined using qRT-PCR. Mean ± SD (*n* = 3). *p < 0.05; **p < 0.01 vs. wild-type mice. **B.** The protein expression levels of hVEGF-A_165_, CYCLIN B1, CDC2, p-CDC2, BRCA-1, MYC, and GADPH in the lung tissues of the mice were determined by western blot analysis. GADPH was used as an internal control.

To validate the gene expression patterns of the three tumorigenesis levels of transgenic mice revealed by microarray analysis, the expression levels of nine selected genes (*hvegf-A_165_*, *cyclin b1*, *cdc2*, *egfr*, *mmp9*, *brca-1*, *myc*, *nrp-1*, and *kdr*) were determined using qRT-PCR (Figure 5A). The expression levels of seven of these genes (*hvegf-A_165_*, *cyclin b1*, *cdc2*, *egfr*, *mmp9*, *nrp-1*, and *kdr*) were significantly higher, verifying the validity of the gene expression patterns that were observed by microarray analysis and IPA. In addition, the expression levels of six proteins in the lung tissues of the three tumorigenesis levels of transgenic mice were evaluated by western blot analysis (Figure 5B). The descending order of protein expression (hVEGF-A_165_, CYCLIN B1, CDC2, p-CDC2, BRCA-1, and MYC) of the different levels of transgenic mice was Tg-level-3 > Tg-level-2 > Tg-level-1 > wild type mice. Of these, the Tg-level-3 transgenic mice exhibited the highest hVEGF-A_165_, CYCLIN B1, CDC2, p-CDC2, BRCA-1, and MYC protein expression levels of all the transgenic mice.

### Endoglin (CD105), fibrogenic genes, and inflammatory cytokines in three tumorigenesis levels of transgenic mice

**Figure 6:**
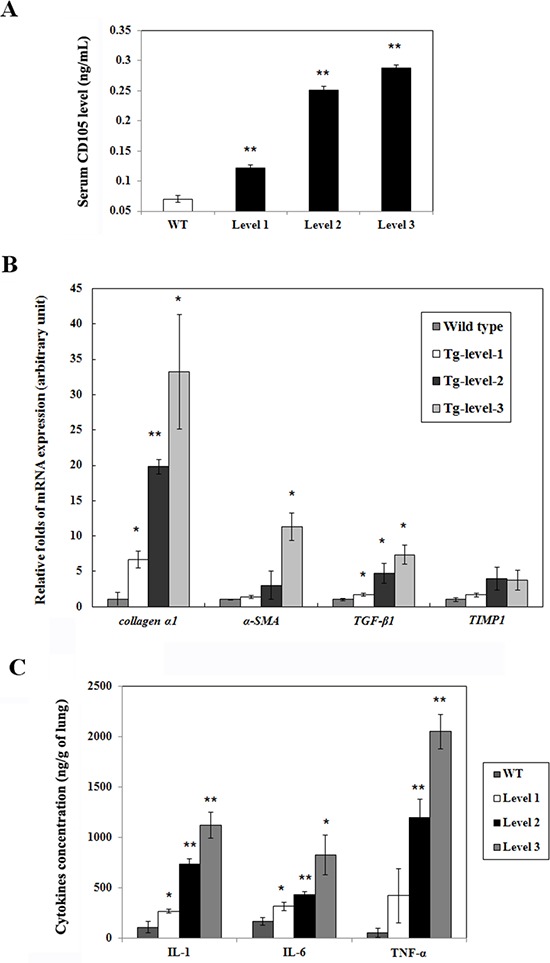
The expression levels of CD105, mRNA, and protein in the lung tissues of wild-type mice and three different tumorigenesis levels of transgenic mice (Tg-level-1, Tg-level-2, and Tg-level-3) **A.** The CD105 level in the serum of the mice was determined using ELISA kit. **p < 0.01 vs. wild-type mice. **B.** The quantitative mRNA expression levels of the fibrogenic genes (*collagen α1*, *α-SMA*, *TGF-β1*, and *TIMP1*) in the lung tissues of the mice were determined using qRT-PCR. Mean ± SD (*n* = 3). *p < 0.05; **p < 0.01 vs. wild-type mice. **C.** The inflammatory cytokines (IL-1, IL-6, and TNF-*α*) in the lung tissues of the mice were determined by ELISA kit. *p < 0.05; **p < 0.01 vs. wild-type mice.

The CD105 level was significantly higher by 1.73-, 3.58-, and 4.10-fold in the Tg-level-1, Tg-level-2, and Tg-level-3 transgenic mice, respectively, compared with that of the wild-type mice (Figure 6A). The increasing order of fibrogenic genes (*collagen α1*, *α-SMA*, *TGF-β1*, and *TIMP1*) of the different levels of transgenic mice was Tg-level-3 > Tg-level-2 > Tg-level-1 > wild-type mice (Figure 6B). In addition, the Tg-level-3 transgenic mice exhibited the serious inflammatory response with the highest inflammatory cytokines (IL-1, IL-6, and TNF-*α*) following by Tg-level-2, Tg-level-1, and wild-type mice (Figure 6C).

### Magnetic resonance imaging (MRI) for monitoring lung tumor growth in transgenic mice

**Figure 7:**
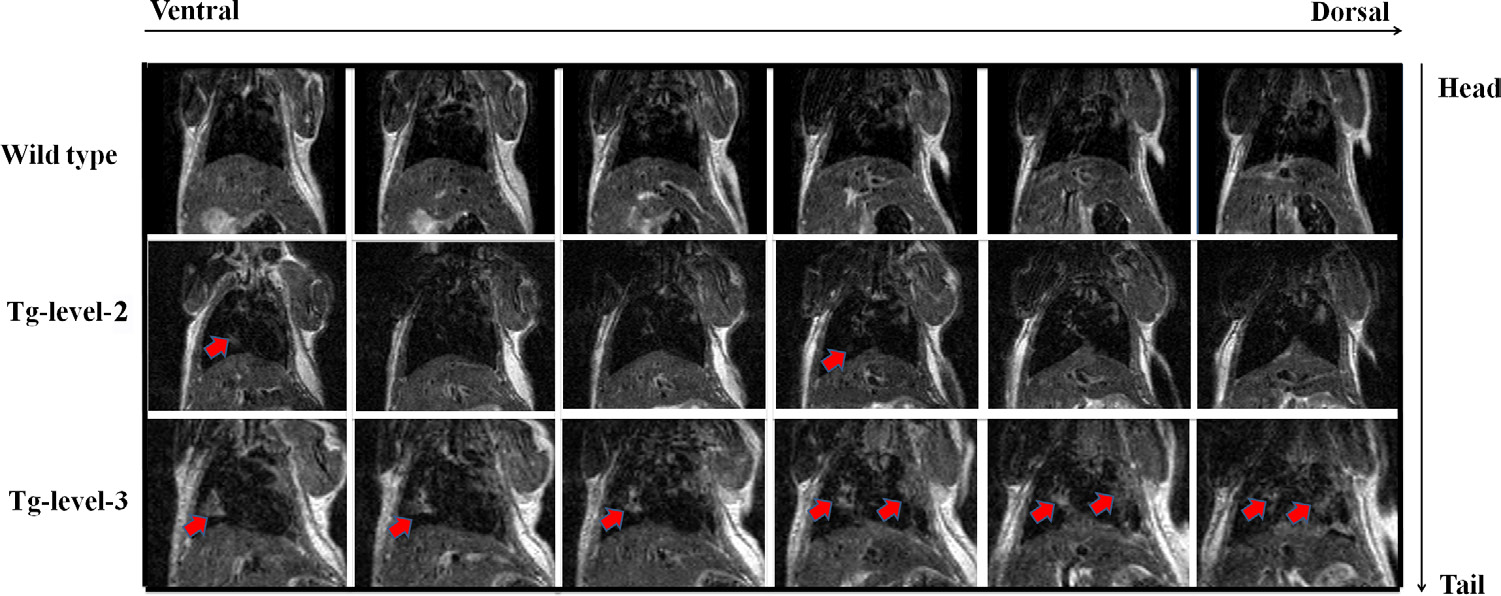
The MRI images of tumors (arrows) in the lung tissues of wild type mice and in two different tumorigenesis levels of transgenic mice (Tg-level-2 and Tg-level-3) Top panel: wild-type mouse. Middle panel: lower hVEGF-A_165_- expressing transgenic mouse (Tg-level-2). Bottom panel: higher hVEGF-A_165_- expressing transgenic mouse (Tg-level-3). Red arrows indicate the solitary nodules of lung tumor.

To evaluate individual tumors *in vivo*, tumors from anesthetized animals were imaged using high-resolution MRI that continuously collected images over a long period of tumor growth and treatment. When the animals became moribund, they were sacrificed, and their lungs were inflated with fixative, embedded in paraffin, and then serially sectioned to compare the tumors using both high-resolution MRI and histological methods. MRI proved to be most useful in detecting lung tumors located in the hilar area and least useful in detecting serosal surface and anterior lobe tumor foci. Figure 7 shows that solitary nodules were observed in four of four recipients in the > 12-month-old, lung-specific hVEGF-A_165_-overexpressing (Tg-level-2 and Tg-level-3) transgenic mice. Histological sections also showed adenocarcinoma foci in the lung; in contrast, no tumors were found in the wild-type mice.

### MicroRNA-16 reduces pulmonary tumorigenesis in VEGF-overexpressing transgenic mice

**Figure 8:**
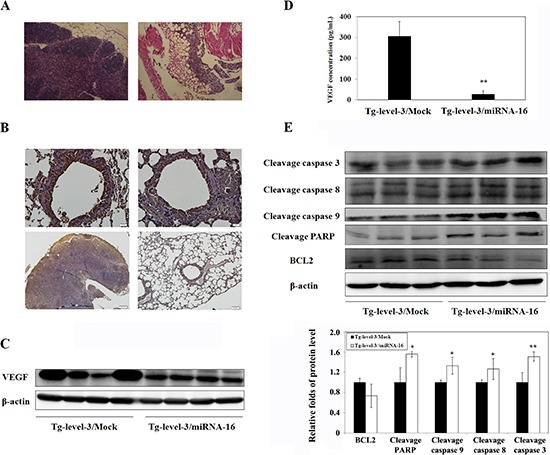
MicroRNA-16 reduces pulmonary tumorigenesis in transgenic mice **A.** Histopathologic sections of lung tissues from Tg-level-3 mice are shown. **B.** The immunohistochemical (IHC) staining of the lung tissues of Tg-level-3 mice is shown. **C.** The protein expression levels of hVEGF-A_165_ and GADPH in the lung tissues of Tg-level-3 mice were determined by western blot analysis. GADPH was used as an internal control. **D.** VEGF concentrations in the serum of Tg-level-3 mice were determined using ELISA. **p < 0.01 vs. Tg-level-3/Mock mice. **E.** The protein expression levels of cleavage caspase 3, cleavage caspase 8, cleavage caspase 9, cleavage PARP, and BCL2 in the lung tissues of Tg-level-3 mice were determined by western blot analyses. β-actin was used as an internal control. *p < 0.05; **p < 0.01 vs. Tg-level-3/Mock mice.

The generated mouse model can be used to investigate the role of VEGF in pulmonary adenocarcinoma. Therefore, we further investigated the inhibitory effect of microRNA-16 (miR-16) on lung tumors (Figure 8) because recent reports have linked the expression of specific microRNAs with tumorigenesis and metastasis. After three intranasal administrations of 20 μg miR-16 or mock miR per mouse once a week, we found that miR-16, which lowered the expression of VEGF in both lung tissues (Figure 8B and 8C) and circulation blood (Figure 8D) (*p* < 0.01), affected the formation of lung tumors in > 12-month-old lung-specific hVEGF-A_165_ overexpressing transgenic mice (Figure 8A). Thus, miR-16 can inhibit lung cancer growth by suppressing VEGF expression. To investigate the molecular mechanisms underlying miR-16-induced apoptosis, apoptosis-related proteins were examined *via* western blot analyses. The results of the western blot analyses revealed that the cleaved forms of caspase 3, 8, 9, and poly (ADP-ribose) polymerase (PARP) were activated after miR-16 treatment in the hVEGF-A_165_ overexpressing transgenic mice (Figure 8E). Based on these results, miR-16 may induce apoptosis *via* both the intrinsic and extrinsic pathways.

### MicroRNA-16 reduces pulmonary tumorigenesis in human orthotopic non-small cell lung cancer xenograft model

**Figure 9:**
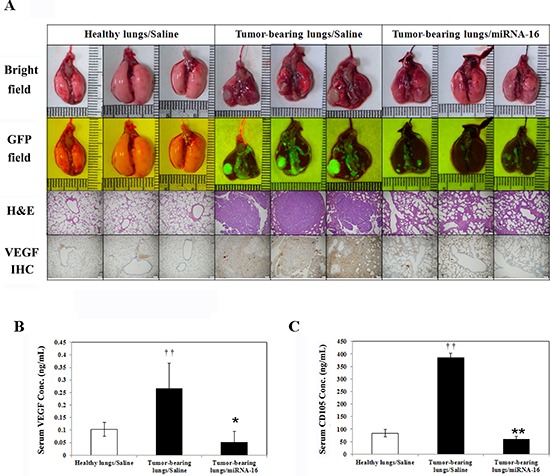
MicroRNA-16 reduces pulmonary tumorigenesis in human orthotopic non-small cell lung cancer xenograft model **A.** Exteriors, histopathologic sections and immunohistochemical (IHC) staining of lung tissue are shown. **B.** VEGF concentrations in the serum of the mice were determined using ELISA. ^††^*p* < 0.01 vs. Healthy lungs/Saline mice. **p < 0.01 vs. Tumor-bearing lungs/Saline mice. **C.** The CD105 level in the serum of the mice was determined using ELISA kit. ^††^*p* < 0.01 vs. Healthy lungs/Saline mice. *p < 0.05 vs. Tumor-bearing lungs/Saline mice.

The therapeutic efficacy of miR-16 was evaluated in the A549-pCAG-iRFP-2A-Verus orthotopic lung tumor mouse model. A549-pCAG-iRFP-2A-Verus tumor cells suspended in matrigel/PBS were injected into the left lung parenchyma to a depth and the wounds were closed with a surgical skin clip. Figure 9A showed that miR-16 lowered pulmonary tumorigenesis *via* decreased the expression of VEGF in both lung tissues (Figure 9A, lower panel) and circulation blood (Figure 9B), and also decreased the expression of CD105 tumor marker in serum (Figure 9C), affected the formation of lung tumors in human orthotopic non-small cell lung cancer xenograft model. Thus, the result was the same with VEGF-overexpressing transgenic mice model that miR-16 can inhibit lung cancer growth by suppressing VEGF expression.

## DISCUSSION

In this study, we developed a method for studying the lung-specific expression of a hVEGF-A_165_ transgene under the control of the mouse Clara cell-specific protein (*mccsp*) gene promoter in > 12-month-old hVEGF-A_165_-overexpressing transgenic mice with significant lung inflammation and adenocarcinoma formation (Figure 2). VEGF increases endothelial cell permeability and enhances endothelial cell migration and proliferation [16], rendering VEGF necessary for tumor growth and angiogenesis [17]. During lung adenocarcinoma development, intrinsic CCSP expression was decreased in the bead-like Clara cells in the lung capillary bronchus of the transgenic mice. Hicks *et al*. [18] also showed that only an occasional cell or segment of the bronchiolar epithelium stained positively for CCSP (also known as CC10) using immunohistochemical methods, and all tumors were found to be uniformly negative for staining. Based on the results of the foregoing studies, it was concluded that the chronic inflammation or serious injuries and even the formation of a tumor during carcinogenesis that occurred in the lung of transgenic mice aged 12 months or older were related to lung-specific VEGF-A_16*5*_ protein expression.

VEGF-A is highly expressed in numerous tumors of the lung, brain and gastrointestinal and urogenital tracts [19]. VEGF-A binds to two receptor tyrosine kinases, VEGFR1 (Flt-1) and VEGFR2 (Flk-1/KDR), and transduces angiogenic signals. This expression is induced through several genetic (activation of oncogenes, or inactivation or loss of tumor suppressor genes) and epigenetic (hypoxia, low pH, interleukin-6, inflammatory cytokines and growth factors such as basic fibroblast growth factor) mechanisms [20]. In NSCLC, VEGF expression is associated with increased tumor microvasculature and potentially poor prognosis [21, 22].

The expression of VEGF-A_165_ is related to the growth and spread of cancer cells. When cancer cells secrete a large amount of VEGF-A_165_, vasculogenesis is induced to provide sufficient nutrition and oxygen to the tumor, which increases the rate of tumor growth [12]. In this study, we confirmed that the expression of VEGF-A_165_ is positively related to the formation of pulmonary cancer (Figure 3A and 3B). VEGF-A_165_ primarily binds to KDR, inducing dimerization of KDR, which promotes intracellular tyrosine phosphorylation that results in a series of signal transductions that promote endothelial cell survival, hyperplasia and differentiation [23].

To examine the molecular mechanisms induced by hVEGF-A_165_ overexpression, we conducted microarray and IPA analyses and found that *cyclin b1* and *cdc2* (cell cycle), *mmp9* (metastasis), and *brca1* and *myc* (oncogenes) were overexpressed in >12-month-old hVEGF-A_165_-overexpressing (Tg-level-2 and Tg-level-3) transgenic mice (Figures 4 and 5). These results suggest that *vegf* overexpression induces cell cycle progression and metastasis by increasing the *cyclin b1*, *cdc2*, and *mmp9* levels. These findings have important implications for the role of VEGF overexpression in pulmonary tumors. CD105 is a membranous protein overexpressed in tumor-associated endothelial cells. In this study, the CD105 level was significantly higher in hVEGF-A_165_-overexpressing transgenic mice than wild-type mice (Figure 6A). In addition, overexpressing hVEGF-A_165_ in Clara cells causes increased fibrogenic genes (Figure 6B) and inflammatory cytokines (Figure 6C) in the lungs of hVEGF-A_165_-overexpressing transgenic mice as compared to wild-type mice. The data presented here strongly suggest that VEGF overexpression may be one of the reasons for the initiation of tumorigenesis. Thus, various VEGF inhibitors are being widely proposed as potential therapeutic agents for cancer treatment. VEGF signaling has become an important target for cancer therapy because of its central role in regulating angiogenesis, and targeting VEGF signaling is proving to be a valuable therapeutic approach in cancer patients. Significantly, the humanized anti-VEGF monoclonal antibody bevacizumab showed promising results when used in combination with chemotherapy in NSCLC phase III trials [24]. However, in another phase III trial, adding bevacizumab to standard chemotherapy did not result in an overall longer survival of the NSCLC patients [25]. Therefore, it is important to identify reliable predictive factors that will allow for the selection of patients who are most likely to benefit from anti-angiogenesis agents in future studies.

Furthermore, the present study provides a method for generating mice for investigating the role of VEGF in pulmonary adenocarcinoma formation. The mice were genetically engineered to specifically express hVEGF-A_165_ in the lung. Furthermore, the mice developed different degrees of tumorigenesis, as demonstrated by histopathological analyses. To evaluate the individual lung tumors *in vivo*, we employed a living image system using high-resolution MRI of anesthetized animals (Figure 7). The MRI technique that was used for the mouse tumor models has several advantages, such as the following: 1) MRI can continuously collect images over a long period of tumor growth and treatment; 2) MRI reveals information ranging from anatomical structure to chemical composition and physiological parameters, such as blood flow [26]; 3) MRI provides much higher contrast between various soft tissues than computed tomography (CT), and paramagnetic contrast agents can further enhance this contrast [27]; and 4) successful applications of MRI in the mouse have led to the development of high-throughput drug discovery techniques [28].

Using the lung-specific adenocarcinoma transgenic mouse model and the A549-pCAG-iRFP-2A-Verus orthotopic lung tumor mouse model, we further investigated the novel approaches for microRNA target therapy and demonstrated that miR-16 effectively inhibits lung cancer growth by suppressing VEGF expression *via* the intrinsic and extrinsic apoptotic pathways (Figure 8).

Few published studies have identified miRNAs that contribute to the expression of VEGF. Hua et al. [29] showed that VEGF is predicted to be targeted by multiple miRNAs, including miR-15b, miR-16, miR-20a and miR-20b, and transfection of these miRNAs into CNE cells (a human nasopharyngeal carcinoma cell line) can inhibit VEGF expression [29]. More recently, Roccaro et al. [30] showed that miR-15a/16 are decreased or completely absent in relapsed/refractory multiple myeloma (MM) patients and that miR-15a/16 are critical regulators of MM pathogenesis. However, strong evidence demonstrating that these miRNAs regulate VEGF in MM and other cancer types has not yet been reported [31, 32].

In conclusion, our hVEGF-A_165_-bearing mice had pronounced pulmonary inflammation, hyperplasia and tumorigenesis that were triggered by higher VEGF-A expression levels in lung-specific Clara cells. We further demonstrated that the intranasal administration of miR-16 could inhibit lung cancer growth by significantly suppressing VEGF expression. Therefore, this animal model may be used to study cancer pathophysiology, tumor-associated inflammation, and the effects of new target therapy interventions.

## MATERIALS AND METHODS

### Detection of the transgene by PCR screening and Southern blot hybridization

The mccsp-Vegf-A_165_-SV40-transgenic mice (FVB) were generated by pronuclear microinjection. A 1,975-bp transgene fragment (Figure 1A), consisting of the *mccsp* promoter, *hVegf-A_165_* cDNA and *SV40 poly(A)* signal sequence, was obtained from the plasmid by *Nru*I-*Sap*I double-digestion. The purified transgene was then microinjected into the male pronuclei of fertilized eggs and transferred to pseudo-pregnant recipient females as previously described [33, 34]. To detect the hVEGF-A_165_ transgene in mice, the pups were rapidly screened by PCR analysis of tail genomic DNA using the primer set hVEGF94(+) and hVEGF315(−), which is listed in Table 1. The screening results were further verified by Southern blot hybridization as previously described [33]. Briefly, 10 μg of genomic DNA were digested with *Nco*I at 37°C overnight, electrophoresed on a 0.8% agarose gel, and then transferred to a Durose nitrocellulose membrane (Stratagene, La Jolla, CA). The *Pst*I-*Bgl*II fragment of the hVEGF-A_165_ cDNA (0.6 kb) was used as a radioactive probe and hybridized to the membrane, and the blots were subjected to autoradiography for one week at −20°C.

**Table 1:** Oligonucleotide primers used for transgene detection, RT-PCR, and real-time PCR analyses

Primer set	Oligonucleotide sequence	Size (bp)	Tm (°C)
hVEGF 94 (+)	5′-AAGGAGGAGGGCAGAATCATC-3′	243	63
hVEGF 315 (−)	5′-GAGGTTTGATCCGCATAATCTG-3′		
cyclin B1 (+)	5′-CCAGAGGCGGATGAGAACA-3′	101	57.8
cyclin B1 (−)	5′-ATGGAGGGTGGGTTGGAAAT-3′		
cdc2 (+)	5′-CACCTGTGGCTGTCAGAGAA-3′	153	57
cdc2 (−)	5′-GGGTAGAGTAAACTCCGTGA-3′		
egfr (+)	5′-GCCATCTGGGTACGTTCAAT-3′	128	58.5
egfr (−)	5′-GCAAGAGGGCAGAATCTGAG-3′		
mmp9 (+)	5′-TGAATCAGCTGGCTTTTGTG-3′	225	53
mmp9 (−)	5′-GTGGATAGCTCGGTGGTGTT-3′		
brca1 (+)	5′-TTCAAGTGCCAGTGTCAAGG-3′	160	56
brca1 (−)	5′-AGGGAGCAAAAGGGAAGAAA-3′		
myc (+)	5′-CTGTCCATTCAAGCAGACGA-3′	113	57.8
myc (−)	5′-TCCAGCTCCTCCTCGAGTTA-3′		
nrp1 (+)	5′-AGCTTCAATGAGCGTCACCT-3′	184	50
nrp1 (−)	5′-CCACCACAGGGTAAGGAGAA-3′		
kdr (+)	5′-GGACCTCAGACTGCAAGGAG-3′	202	57
kdr (−)	5′-TCTTGGAGGACAGAGCCACT-3′		
β-actin (+)	5′-ACACCCGCCACCAGTTCGC-3′	164	65
β-actin (−)	5′-ACCATTCCCACCATCACAC-3′		
collagen α1 (+)	5′-CCAAGGGTAACAGTGGTGAA-3′	149	57
collagen α1 (−)	5′-CCTCGTTTTCCTTCTTCTCCG-3′		
α-SMA (+)	5′-GGCTCTGGGCTCTGTAAGG-3′	124	57
α-SMA (−)	5′-CTCTTGCTCTGGGCTTCATC-3		
TGF-β1 (+)	5′-GGTTCATGTCATGGATGGTGC-3′	170	59
TGF-β1 (−)	5′-TGACGTCACTGGAGTTGTACGG-3′		
TIMP1 (+)	5′-GCATCTCTGGCATCTGGCATC-3′	214	61
TIMP1 (−)	5′-GCGGTTCTGGGACTTGTGGGC-3′		

### Histology and immunohistochemistry

Lung tissue was fixed in 10% buffered formaldehyde and processed for histological examination by hematoxylin and eosin (H&E) staining [35, 36]. Formalin-fixed and paraffin-embedded samples were cut into 5-μm-thick sections, deparaffinized and rehydrated through an alcohol-to-water gradient, and then treated with boiling water for 15 min. The sections were incubated in 3% hydrogen peroxide for 10 min to block endogenous peroxidase activity and then incubated overnight at 4°C with the primary rabbit monoclonal antibody hVEGF-A at a 1:40 working dilution. For antigen retrieval, the sections were immunostained using the VECTASTAIN^®^ ABC kit (UNIVERSAL, VECTOR, USA) in accordance with the manufacturer's specifications. Diaminobenzidine (DAB) was used for staining development, and the sections were counterstained with hematoxylin. Negative controls consisted of substituting normal serum for primary antibodies.

### Western blot analysis

Western blot analysis of proteins expressed in lung tissue was performed. Lung tissues were homogenized in 500 μl of RIPA buffer (5 mM Tris–HCl pH 7.4, 0.15 M NaCl, 1% NP40, 0.25% sodium deoxycholate, 5 mM EDTA and 1 mM ethylene glycol-bis (2-aminoethyl-ether)-N, N, N, N-tetraacetic acid), and the homogenates were centrifuged at 12,000 × *g* for 30 min at 4°C. Then, 40 μg of protein was separated by 10%, 12%, or 13% SDS-PAGE and electrotransferred to polyvinylidene difluoride membranes [37, 38]. The membranes were incubated in blocking solution at room temperature for 2 h and then with primary antibody (VEGF-A, Cyclin B1, CDC2, pCDC2, BRCA-1, and GAPDH; cleavage caspase 3, cleavage caspase 8, cleavage caspase 9, cleavage PARP, BCL2, and β-actin) for 2 h. After washing, the membranes were incubated with a goat anti-rabbit or goat anti-mouse IgG peroxidase-conjugated secondary antibody directed against the primary antibody, and the membranes were developed using an enhanced chemiluminescence western blot detection system [39].

### Chick embryo chorioallantoic membrane assay

Angiogenic activity in chick embryo chorioallantoic membranes (CAMs), which have been widely used to study angiogenesis, was evaluated [40]. Eggs were incubated in an incubator at 38°C with 60% humidity. A small window was made in the shell on day 3 of chick embryo development under aseptic conditions. The window was then resealed with adhesive tape, and the eggs were returned to the incubator until day 5 of chick embryo development. On day 5, wild type and three different tumorigenesis levels of lung tissues (Tg-level-1, Tg-level-2, and Tg-level-3) were placed on top of the CAM, and the eggs were resealed and returned to the incubator for 48 h and 72 h, which was equivalent to day 7 and day 9, respectively, of chick embryo development.

### Microarray analysis and ingenuity pathway analysis (IPA)

Total RNA was extracted from lung tissue using Trizol reagent (Invitrogen, Carlsbad, CA) according to the manufacturer's instructions [41]. Total RNA (0.5 μg) from transgenic (Tg-level-3) and wild-type mice was labeled with Cy3- and Cy5-conjugated dCTP using a reverse transcription reaction, respectively, and the labeled cDNA mixture was concentrated using an EtOH precipitation method. The concentrated Cy3- or Cy5-labeled cDNA was mixed and then placed on an Agilent mouse whole genome 44K oligo microarray and placed in the Agilent microarray hybridization chamber. The fluorescent image of the hybridized microarray was scanned using an Agilent microarray scanner and analyzed using Agilent Feature Extraction software. The identified genes (Cy5/Cy3 signal ratios greater than 2.0) were imported into IPA software (Ingenuity Systems, Redwood, CA), which is a commercially available software for evaluating microarray data.

### Semi-quantitative RT-PCR and real-time RT-PCR

Two micrograms of total RNA from lung tissue were resuspended in 9 μl of diethylpyrocarbonate (DEPC)-treated water and used to synthesize first-strand cDNA using random primers and ImProm-II™ reverse transcriptase in a total volume of 20 μl at 40°C for 1 h. To validate the gene expression identified, RT-PCR and qRT-PCR were performed on nine genes (*vegf*, *cyclin b1*, *cdc2*, *egfr*, *mmp9*, *brca-1*, *myc*, *nrp-1*, and *kdr*) in the IPA analysis and four fibrogenic genes (*collagen α1*, *α-SMA*, *TGF-β1*, and *TIMP1*) using cDNA from lung tissue [42]. The cDNA sequence of *β-actin* was used as an internal control. For further PCR amplification, an aliquot (1/10) of the RT product was adjusted to contain 0.1 μg of each primer, and additional buffer was added to a total volume of 20 μl. RT-PCR was performed using a Thermal Cycler 2720, and qRT-PCR was analyzed using the SYBR Green method and Rotor-Gene™ 6000.

### Enzyme-linked immunosorbent assay (ELISA)

We performed ELISA of IL-1, IL-6, TNF-α (all purchased from eBioscience, San Diego, CA), CD105 (R&D Systems, Minneapolis, MN), and VEGF (PeproTech, Rocky Hill, NJ) according to the manufacturer's protocol.

### Magnetic resonance imaging (MRI)

We conducted MRI measurements using a 1T desktop magnetic resonance scanner (Aspect M2 Compact High Performance MR System). We anesthetized the mice with 1% isoflurane in an oxygen-air mixture, and then imaged the mice using a gradient echo fast imaging sequence with respiratory and cardiac gating in both the coronal and axial planes. A butterfly cannula filled with heparinized saline solution was inserted into the tail vein and left in place. T2 weighted imaging sequences were obtained with the following parameters: T2 SE: TR 2000–2500 ms, TE 15, 30, 45, 60 ms (2T), and TE 20, 40, 60, 80 ms (1T). MRIs were obtained with a field of view (FOV) of 3 × 3 cm (2T) or 6 × 6 cm (1T), a matrix of 128 × 128 or 200 × 200 pixels, and a slice thickness of 1 to 2 mm.

### MicroRNA-16 reduces pulmonary tumorigenesis in vascular endothelial growth factor (VEGF)-overexpressing transgenic mice

The transgenic mice (Tg-level-3) were given a standard laboratory diet and distilled water *ad libitum* and kept on a 12-h light/dark cycle at 22 ± 2°C. This study was conducted according to institutional guidelines and approved by the Institutional Animal Care and Utilization Committee of National Chung Hsing University, Taiwan (IACUC-98-3). For *in vivo* transfection, *in vivo*-jetPEI TM transfection reagent (Polyplus Transfection, Illkirch, France) was used following the manufacturer's protocol. The transgenic mice were randomly assigned to the following two groups for treatment: Tg/Mock (*in vivo*-jetPEI in 5% glucose solution) and Tg/miR-16 (miR-16 mixed with *in vivo*-jetPEI in 5% glucose solution). MicroRNA-16 (3′-UTR 793–822), obtained from Thermo Scientific (Hudson, NH, USA), was intranasally administered three times at 20 μg per mouse once a week [43]. The mice were then sacrificed after four weeks of miR-16 administration, and lung tissues were collected for pathological histology, immunohistochemistry staining, and protein extraction.

### MicroRNA-16 reduces pulmonary tumorigenesis in human orthotopic non-small cell lung cancer xenograft model

A549 cells were transfected with pCAG-iRFP-2A-Verus, which have been validated as stable clones. piRFP was obtained from Dr. Vladislav V. Verkhusha (Albert Einstein College of Medicine, USA) and pCAG-2A-Verus were provided by Prof. Hong-Lin Su (Department of Life Sciences, National Chung Hsing University, Taiwan). For orthotopic tumor implantation, male nude (nu/nu) mice were anesthetized and A549 tumor cells (2.5 × 10^6^ per mice) suspended in matrigel/PBS were injected into the lung parenchyma [44]. All the nude mice develop lung tumors at 6 weeks after intrapulmonary injection of tumor cells. The mice were then sacrificed after miR-16 administration, and lung tissues and sera were collected for pathological histology, immunohistochemistry staining, serum VEGF and CD105 detections.

### Statistical analysis

The experimental values are expressed as the mean ± standard deviation (SD). All data were analyzed using *t*-test, and statistical significance was presented as *p* < 0.05 (*) or *p* < 0.01 (**).

## SUPPLEMENTARY TABLE


