# Supplementary material for: Lung tumorigenesis induced by human vascular endothelial growth factor (hVEGF)-A165 overexpression in transgenic mice and amelioration of tumor formation by miR-16
Source: Oncotarget. 2015 Apr 22;6(12):10222–38. doi: 10.18632/oncotarget.3390 (PMC4496351; doi:10.18632/oncotarget.3390)
Supplement: Supplementary file 1 [file oncotarget-06-10222-s001.pdf]

SUPPLEMENTARY TABLE

Supplementary Table S1. At least 2-fold differentially expressed genes, of which 580 genes (cell-cell adhesion: 80, signal pathway: 532, apoptosis: 53, and cell cycle regulation: 477), are up-regulated in the lung tissue of Tg-level-3 mice compared to the lung tissue of wild type mice by cDNA microarray

Genbank Accession No.	Gene Symbol	Normalized Expression level (fold)	Description
NM_023209	Pbk	45.48	PDZ binding kinase
AJ237585	Ncapg	43.10	on-SMC condensin I complex, subunit G
NM_012012	Exo1	42.32	exonuclease 1
NM_009689	Birc5	34.70	baculoviral IAP repeat-containing 5
NM_008234	Hells	29.32	helicase, lymphoid specific
NM_012025	Racgap1	27.34	Rac GTPase-activating protein 1
NM_146235	Ercc6l	26.59	excision repair cross-complementing rodent repair
NM_028232	Sgol1	25.99	shugoshin-like 1
NM_010931	Uhrf1	25.73	ubiquitin-like, containing PHD and RING finger domains, 1
NM_026410	Cdca5	24.85	cell division cycle associated 5
NM_009772	Bub1	23.96	budding uninhibited by benzimidazoles 1 homolog
NM_021891	Fignl1	23.89	fidgetin-like 1
NM_011634	Traip	23.49	TRAF-interacting protein
X82786	Mki67	21.76	antigen identified by monoclonal antibody Ki 67
NM_010931	Uhrf1	21.28	ubiquitin-like, containing PHD and RING finger domains, 1
NM_011623	Top2a	19.89	topoisomerase (DNA) II alpha
Y09632	Kif20a	19.30	kinesin family member 20A
NM_008567	Mcm6	18.89	minichromosome maintenance deficient 6
X82786	Mki67	18.87	antigen identified by monoclonal antibody Ki 67
NM_007659	Cdc2a	18.62	cell division cycle 2 homolog A
NM_026515	2810417H13Rik	18.50	RIKEN cDNA 2810417H13 gene
BC050071	2610036L11Rik	18.39	RIKEN cDNA 2610036L11 gene
NM_021886	Cenph	18.12	centromere protein H
NM_183089	2600005O03Rik	18.08	RIKEN cDNA 2600005O03 gene
NM_172598	Wdhd1	17.69	WD repeat and HMG-box DNA binding protein 1
NM_013538	Cdca3	17.65	cell division cycle associated 3
AK045323	Oip5	17.64	Opa interacting protein 5
NM_028109	Tpx2	17.24	TPX2, microtubule-associated protein homolog
NM_009004	Kif20a	17.24	kinesin family member 20A

(*Continued*)

Genbank Accession No.	Gene Symbol	Normalized Expression level (fold)	Description
NM_178183	Hist1h2ak	17.24	histone cluster 1, H2ak
NM_026560	Cdca8	16.85	cell division cycle associated 8
NM_011126	Plunc	16.72	palate, lung, and nasal epithelium carcinoma associated
NM_009828	Ccna2	16.61	cyclin A2
NM_027290	Mcm10	16.49	minichromosome maintenance deficient 10
AK010336	Trip13	16.26	thyroid hormone receptor interactor 13
AK049676	Cenpe	16.26	centromere protein E
NM_145588	Kif22	15.97	kinesin family member 22
NM_178182	Hist1h2ai	15.91	histone cluster 1, H2ai
AK028715	AI662270	15.65	expressed sequence AI662270
NM_009104	Rrm2	15.13	ribonucleotide reductase M2
NM_010766	Marco	14.91	macrophage receptor with collagenous structure
NM_025415	Cks2	14.75	CDC28 protein kinase regulatory subunit 2
NM_011234	Rad51	14.53	RAD51 homolog
NM_133851	Nusap1	14.44	nucleolar and spindle associated protein 1
NM_177420	Psat1	14.44	phosphoserine aminotransferase 1
NM_178683	Depdc1b	14.22	DEP domain containing 1B
NM_008522	Ltf	14.21	lactotransferrin
NM_008522	Ltf	14.08	lactotransferrin
NM_010849	Myc	13.91	myelocytomatosis oncogene
NM_011121	Plk1	13.86	polo-like kinase 1
NM_175661	Hist1h2af	13.73	histone cluster 1, H2af
NM_025565	Spc25	13.54	SPC25, NDC80 kinetochore complex component, homolog
NM_010849	Myc	13.17	myelocytomatosis oncogene
AK018691	Gramd3	13.15	GRAM domain containing 3
NM_007630	Ccnb2	13.12	cyclin B2
NM_009773	Bub1b	13.06	budding uninhibited by benzimidazoles 1 homolog, beta
NM_023284	Nuf2	12.95	NUF2, NDC80 kinetochore complex component, homolog
NM_007900	Ect2	12.88	ect2 oncogene
NM_016966	Phgdh	12.79	3-phosphoglycerate dehydrogenase
NM_025415	Cks2	12.55	CDC28 protein kinase regulatory subunit 2
NM_016966	Phgdh	12.50	3-phosphoglycerate dehydrogenase
NM_023223	Cdc20	11.83	cell division cycle 20 homolog

(*Continued*)

Genbank Accession No.	Gene Symbol	Normalized Expression level (fold)	Description
NM_021718	Ms4a4b	11.73	membrane-spanning 4-domains, subfamily A, member 4B
NM_024184	Asf1b	11.70	ASF1 anti-silencing function 1 homolog B
NM_026560	Cdca8	11.66	cell division cycle associated 8
NM_011677	Ung	11.62	uracil DNA glycosylase
M19902		11.62	immunoglobulin heavy chain (V10 family)
NM_009862	Cdc45l	11.56	cell division cycle 45 homolog-like
NM_026240	Gramd3	11.45	GRAM domain containing 3
NM_026014	Cdt1	11.41	chromatin licensing and DNA replication factor 1
NM_009764	Brca1	11.39	breast cancer 1
NM_016904	Cks1b	11.38	CDC28 protein kinase 1b
NM_025759	Speer4d	11.20	spermatogenesis associated glutamate (E)-rich protein 4d
NM_029766	Dtl	11.11	denticleless homolog
NM_008638	Mthfd2	10.69	methylenetetrahydrofolate dehydrogenase (NAD+ dependent)
XM_129658	Cenpf	10.63	centromere protein F
NM_007681	Cenpa	10.27	centromere protein A
NM_146256	Hpdl	10.26	4-hydroxyphenylpyruvate dioxygenase-like
NM_001013811	EG434197	10.21	predicted gene
NM_021790	Cenpk	10.07	centromere protein K
NM_016966	Phgdh	10.07	3-phosphoglycerate dehydrogenase
BF720745		9.72	phosphoinositide-3-kinase, regulatory subunit 5, p101
NM_021319	Pglyrp2	9.71	peptidoglycan recognition protein 2
NM_011132	Pole	9.66	polymerase (DNA directed), epsilon
NM_175449	A630077B13Rik	9.60	RIKEN cDNA A630077B13 gene
NM_019499	Mad2l1	9.59	MAD2 (mitotic arrest deficient, homolog)-like 1 (yeast)
BC049694	Cdkn3	9.55	cyclin-dependent kinase inhibitor 3
NM_175177	Bdh1	9.52	3-hydroxybutyrate dehydrogenase, type 1
NM_009387	Tk1	9.07	thymidine kinase 1
U83902	Mad2l1	9.06	MAD2 (mitotic arrest deficient, homolog)-like 1 (yeast)
NM_152839	Igj	9.00	immunoglobulin joining chain
NM_008566	Mcm5	8.88	minichromosome maintenance deficient 5, cell division cycle 46
NM_011497	Aurka	8.86	aurora kinase A

(*Continued*)

Genbank Accession No.	Gene Symbol	Normalized Expression level (fold)	Description
NM_026785	Ube2c	8.82	ubiquitin-conjugating enzyme E2C
NM_181416	Arhgap11a	8.64	Rho GTPase activating protein 11A
NM_026024	Ube2t	8.62	ubiquitin-conjugating enzyme E2T
NM_025626	3110001A13Rik	8.49	RIKEN cDNA 3110001A13 gene
NM_010790	Melk	8.46	maternal embryonic leucine zipper kinase
NM_145150	Prc1	8.39	protein regulator of cytokinesis 1
NM_007799	Ctse	8.28	cathepsin E
AK013116	Gins1	8.27	GINS complex subunit 1 (Psf1 homolog)
NM_009830	Ccne2	8.22	cyclin E2
NM_010391	H2-Q10	7.93	histocompatibility 2, Q region locus 10
NM_026282	Spc24	7.90	SPC24, NDC80 kinetochore complex component, homolog
NM_007651	Cd53	7.90	CD53 antigen
NM_026862	Cd177	7.83	CD177 antigen
NM_011867	Slc26a4	7.78	solute carrier family 26, member 4
NM_173867	Rcc2	7.71	regulator of chromosome condensation 2
NM_207161	BC048355	7.67	cDNA sequence BC048355
NM_152839	Igj	7.33	immunoglobulin joining chain
NM_178309	Brip1	7.32	BRCA1 interacting protein C-terminal helicase 1
NM_010892	Nek2	7.32	NIMA (never in mitosis gene a)-related expressed kinase 2
BC027063	Bdh1	7.25	3-hydroxybutyrate dehydrogenase, type 1
NM_007633	Ccne1	7.23	cyclin E1
NM_029803	Ifi27	7.21	interferon, alpha-inducible protein 27
NM_001004140	Ckap2	7.17	cytoskeleton associated protein 2
NM_133774	Stard4	7.13	StAR-related lipid transfer (START) domain containing 4
NM_021288	Tyms	7.11	thymidylate synthase
NM_011260	Reg3g	7.09	regenerating islet-derived 3 gamma
NM_021288	Tyms	7.04	thymidylate synthase
NM_011337	Ccl3	6.92	chemokine (C-C motif) ligand 3
BC004786	Igh	6.83	immunoglobulin heavy chain complex
NM_011404	Slc7a5	6.75	solute carrier family 7 (cationic amino acid transporter, y+ system), member 5
NM_198620	Gm440	6.67	gene model 440
X06342	Spink3	6.64	serine peptidase inhibitor, Kazal type 3
NM_028230	Shmt2	6.60	serine hydroxymethyltransferase 2 (mitochondrial)

(*Continued*)

Genbank Accession No.	Gene Symbol	Normalized Expression level (fold)	Description
NM_025495	Cenpp	6.59	centromere protein P
NM_018868	Nol5	6.57	nucleolar protein 5
NM_027411	Ccdc99	6.56	coiled-coil domain containing 99
AK038257	A130090K04Rik	6.45	RIKEN cDNA A130090K04 gene
NM_029523	Depdc1a	6.45	DEP domain containing 1a
BC036300	1700054N08Rik	6.39	RIKEN cDNA 1700054N08 gene
NM_029752	Bri3bp	6.37	Bri3 binding protein
NM_080850	Pask	6.33	PAS domain containing serine/threonine kinase
AK011422	Nup43	6.27	nucleoporin 43
NM_019501	Pdss1	6.24	prenyl (solanesyl) diphosphate synthase, subunit 1
M30774	Tyms-ps	6.24	thymidylate synthase, pseudogene
NM_025626	3110001A13Rik	6.16	RIKEN cDNA 3110001A13 gene
NM_183224	7530404M11Rik	6.16	RIKEN cDNA 7530404M11 gene
BF578595		6.07	V(kappa) gene product
NM_016723	Uchl3	6.06	ubiquitin carboxyl-terminal esterase L3 (ubiquitin thiolesterase)
NM_013898	Timm8a1	6.04	translocase of inner mitochondrial membrane 8 homolog a1 (yeast)
NM_007550	Blm	6.03	bloom syndrome homolog (human)
NM_010049	Dhfr	5.94	dihydrofolate reductase
NM_001013368	E2f8	5.89	E2F transcription factor 8
NM_144958	Eif4a1	5.71	eukaryotic translation initiation factor 4A1
NM_009272	Srm	5.64	spermidine synthase
NM_015811	Rgs1	5.63	regulator of G-protein signaling 1
NM_007832	Dck	5.58	deoxycytidine kinase
BC089618	OTTMUSG00000000971	5.56	predicted gene, OTTMUSG00000000971
NM_133774	Stard4	5.54	StAR-related lipid transfer (START) domain containing 4
NM_029752	Bri3bp	5.53	Bri3 binding protein
NM_029797	Mnd1	5.53	meiotic nuclear divisions 1 homolog (S. cerevisiae)
NM_008449	Kif5c	5.49	kinesin family member 5C
NM_019698	Aldh18a1	5.47	aldehyde dehydrogenase 18 family, member A1
NM_010849	Myc	5.46	myelocytomatosis oncogene
L32836	Ahcy	5.42	S-adenosylhomocysteine hydrolase
NM_009687	Apex1	5.41	apurinic/apyrimidinic endonuclease 1
NM_013880	Plcl2	5.36	phospholipase C-like 2
NM_053110	Gpnmb	5.33	glycoprotein (transmembrane) nmb

(*Continued*)

Genbank Accession No.	Gene Symbol	Normalized Expression level (fold)	Description
NM_008704	Nme1	5.28	expressed in non-metastatic cells 1, protein
NM_008021	Foxm1	5.27	forkhead box M1
AK005695	Speer5-ps1	5.27	spermatogenesis associated glutamate (E)-rich protein 5, pseudogene 1
NM_010479	Hspa1a	5.26	heat shock protein 1A
NM_010699	Ldha	5.22	lactate dehydrogenase A
BC015304	Ahcy	5.08	S-adenosylhomocysteine hydrolase
NM_009363	Tff2	5.08	trefoil factor 2 (spasmolytic protein 1)
NM_009171	Shmt1	5.06	serine hydroxymethyltransferase 1
NM_013538	Cdca3	5.05	cell division cycle associated 3
NM_020258	Slc37a2	5.03	solute carrier family 37, member 2
NM_011081	Piga	5.02	phosphatidylinositol glycan anchor biosynthesis, class A
NM_026438	Ppa1	5.02	pyrophosphatase (inorganic) 1
NM_178792	Sirpb1	5.00	signal-regulatory protein beta 1
NM_153418	U46068	4.98	cDNA sequence U46068
NM_144526	6720460F02Rik	4.96	RIKEN cDNA 6720460F02 gene
NM_023203	2410015N17Rik	4.92	RIKEN cDNA 2410015N17 gene
NM_133900	Psph	4.92	phosphoserine phosphatase
NM_010479	Hspa1a	4.80	heat shock protein 1A
NM_029752	Bri3bp	4.79	Bri3 binding protein
NM_133900	Psph	4.78	phosphoserine phosphatase
BC053747	Slc43a1	4.74	solute carrier family 43, member 1
L26316	Dhfr	4.69	dihydrofolate reductase
BC034056	Dsg2	4.67	desmoglein 2
AI323028		4.66	thymidylate synthase
NM_176835	2810451A06Rik	4.64	RIKEN cDNA 2810451A06 gene
AK049387	Rps24	4.58	ribosomal protein S24
NM_028595	Ms4a6c	4.57	membrane-spanning 4-domains, subfamily A, member 6C
BC082565	4833427B12Rik	4.57	RIKEN cDNA 4833427B12 gene
NM_023058	Pkmyt1	4.49	protein kinase, membrane associated tyrosine/threonine 1
NM_028177	Ndufab1	4.47	NADH dehydrogenase (ubiquinone) 1, alpha/beta subcomplex, 1
XM_484782	EG433225	4.46	predicted gene, EG433225
NM_010477	Hspd1	4.42	heat shock protein 1 (chaperonin)

(*Continued*)

Genbank Accession No.	Gene Symbol	Normalized Expression level (fold)	Description
NM_027238	1810054D07Rik	4.36	RIKEN cDNA 1810054D07 gene
BC049694	Cdkn3	4.35	cyclin-dependent kinase inhibitor 3
NM_010699	Ldha	4.34	lactate dehydrogenase A
NM_198652	6430706D22Rik	4.28	RIKEN cDNA 6430706D22 gene
NM_172451	Galnt6	4.25	UDP-N-acetyl-alpha-D-galactosamine:polypeptide N-acetylgalactosaminyltransferase 6
AK048051	Spcs3	4.25	signal peptidase complex subunit 3 homolog
NM_008303	Hspe1	4.24	heat shock protein 1 (chaperonin 10)
NM_021788	Sap30	4.24	sin3 associated polypeptide
NM_183249	1100001G20Rik	4.24	RIKEN cDNA 1100001G20 gene
NM_019670	Diap3	4.22	diaphanous homolog 3 (Drosophila)
NM_009698	Aprt	4.18	adenine phosphoribosyl transferase
NM_022324	Sdf2l1	4.16	stromal cell-derived factor 2-like 1
NM_027061	Zpbp2	4.15	zona pellucida binding protein 2
NM_007995	Fcna	4.14	ficolin A
NM_009400	Tnfrsf18	4.13	tumor necrosis factor receptor superfamily, member 18
NM_031195	Msr1	4.12	macrophage scavenger receptor 1
NM_025675	Atpbd4	4.06	ATP binding domain 4
X67198		4.05	isolate 1B4 immunoglobulin light chain variable region (Igk-V)
NM_007628	Ccna1	4.02	cyclin A1
NM_001013367	Prkaa1	4.01	protein kinase, AMP-activated, alpha 1 catalytic subunit
NM_145632	Polr2h	4.00	polymerase (RNA) II (DNA directed) polypeptide H
BF577927		3.99	immunoglobulin kappa chain variable 1 (V1)
NM_013689	Tec	3.98	cytoplasmic tyrosine kinase, Dscr28C related (Drosophila)
M17518		3.97	lactate dehydrogenase A-4 pseudogene mRNA, 3' end
NM_172713	Sdad1	3.97	SDA1 domain containing 1
NM_007633	Ccne1	3.97	cyclin E1
NM_026023	Nudcd2	3.97	NudC domain containing 2
NM_013917	Pttg1	3.95	pituitary tumor-transforming 1
NM_023587	Ptplb	3.94	protein tyrosine phosphatase-like (proline instead of catalytic arginine), member b
AK008094	Igl-V1	3.93	immunoglobulin lambda chain, variable 1
NM_007388	Acp5	3.92	acid phosphatase 5, tartrate resistant

(*Continued*)

Genbank Accession No.	Gene Symbol	Normalized Expression level (fold)	Description
NM_027954	Syce2	3.92	synaptonemal complex central element protein 2
NM_010477	Hspd1	3.90	heat shock protein 1 (chaperonin)
NM_174960	Gimap9	3.90	GTPase, IMAP family member 9
NM_010479	Hspa1a	3.89	heat shock protein 1A
AK048355	Atad5	3.88	ATPase family, AAA domain containing 5
NM_025558	Cyb5b	3.85	cytochrome b5 type B
NM_145126	Chi3l4	3.83	chitinase 3-like 4
NM_027652	D5Wsu178e	3.82	DNA segment, Chr 5, Wayne State University 178, expressed
NM_007403	Adam8	3.81	a disintegrin and metallopeptidase domain 8
NM_031195	Msr1	3.79	macrophage scavenger receptor 1
AK083148	Gm71	3.75	gene model 71, (NCBI)
AF206026		3.75	immunoglobulin kappa chain variable 28 (V28)
BC048546	BC048546	3.69	cDNA sequence BC048546
NM_009139	Ccl6	3.68	chemokine (C-C motif) ligand 6
NM_145211	Oas1a	3.68	2'-5' oligoadenylate synthetase 1A
NM_175507	Tmem20	3.67	transmembrane protein 20
NM_013689	Tec	3.66	cytoplasmic tyrosine kinase, Dscr28C related (Drosophila)
NM_134038	Slc16a6	3.66	solute carrier family 16 (monocarboxylic acid transporters), member 6
NM_020258	Slc37a2	3.65	solute carrier family 37 (glycerol-3-phosphate transporter), member 2
AK012387	1190002F15Rik	3.61	RIKEN cDNA 1190002F15 gene
NM_197889	Ifnz	3.59	interferon zeta
NM_203507	Rwdd4a	3.57	RWD domain containing 4A
NM_023125	Kng1	3.57	kininogen 1
AF152371		3.55	immunoglobulin kappa chain variable 28 (V28)
NM_011055	Pde3b	3.55	phosphodiesterase 3B, cGMP-inhibited
NM_009192	Sla	3.55	src-like adaptor
NM_054098	Steap4	3.54	STEAP family member 4
NM_025615	2810004N23Rik	3.53	RIKEN cDNA 2810004N23 gene
NM_008491	Lcn2	3.53	lipocalin 2
NM_138677	Edem1	3.50	ER degradation enhancer, mannosidase alpha-like 1
NM_021383	Rqcd1	3.49	rcd1 (required for cell differentiation) homolog 1 (S. pombe)
NM_020509	Retnla	3.49	resistin like alpha

(*Continued*)

Genbank Accession No.	Gene Symbol	Normalized Expression level (fold)	Description
XM_138369		3.49	immunoglobulin heavy variable V1-66
XM_358068		3.48	predicted: *mus musculus* gene model 1502, (NCBI) (Gm1502), mRNA
NM_007894	Ear1	3.48	eosinophil-associated, ribonuclease A family, member 1
NM_013658	Sema4a	3.48	sema domain , (semaphorin) 4A
NM_027945	Csl	3.47	citrate synthase like
NM_010401	Hal	3.46	histidine ammonia lyase
NM_009471	Umps	3.44	uridine monophosphate synthetase
NM_008466	Kpna3	3.44	karyopherin (importin) alpha 3
NM_027654	Pcgf6	3.44	polycomb group ring finger 6
NM_013532	Lilrb4	3.41	leukocyte immunoglobulin-like receptor, subfamily B, member 4
NM_172722	C330023M02Rik	3.40	RIKEN cDNA C330023M02 gene
NM_013559	Hsp110	3.40	heat shock protein 110
NM_133771	Memo1	3.39	mediator of cell motility 1
NM_020509	Retnla	3.37	resistin like alpha
NM_009728	Atp10a	3.37	ATPase, class V, type 10A
NM_031254	Trem2	3.37	triggering receptor expressed on myeloid cells 2
NM_010864	Myo5a	3.37	myosin Va
NM_001013026	Ttf2	3.36	transcription termination factor, RNA polymerase II
XM_486435		3.35	predicted: *mus musculus* similar to immunoglobulin light chain variable region (LOC434586)
NM_025566	Tnfaip8l1	3.34	tumor necrosis factor, alpha-induced protein 8-like 1
NM_145153	Oas1f	3.34	2′-5′ oligoadenylate synthetase 1F
NM_008404	Itgb2	3.34	integrin beta 2
AK004515	1190007I07Rik	3.34	RIKEN cDNA 1190007I07 gene
AY173987		3.33	predicted gene, EG668469
NM_133771	Memo1	3.32	mediator of cell motility 1
AK019130	Trfp	3.31	Trf (TATA binding protein-related factor)-proximal protein homolog (Drosophila)
XM_132528		3.31	predicted: *mus musculus* replication factor C (activator 1) 3 (Rfc3)
XM_487249		3.30	predicted: *mus musculus* similar to monoclonal antibody heavy chain (LOC435333)
NM_010168	F2	3.30	coagulation factor II
NM_053111	Ear6	3.29	eosinophil-associated, ribonuclease A family, member 6

(*Continued*)

Genbank Accession No.	Gene Symbol	Normalized Expression level (fold)	Description
NM_001024700		3.29	*mus musculus* similar to immunoglobulin heavy chain variable region (LOC238447)
NM_138599	Tomm70a	3.29	translocase of outer mitochondrial membrane 70 homolog A (yeast)
AK005011	Proz	3.28	protein Z, vitamin K-dependent plasma glycoprotein
NM_053111	Ear6	3.28	eosinophil-associated, ribonuclease A family, member 6
NM_008243	Mst1	3.28	macrophage stimulating 1 (hepatocyte growth factor-like)
NM_008188	Thumpd3	3.28	THUMP domain containing 3
NM_009731	Akr1b7	3.27	aldo-keto reductase family 1, member B7
NM_016895	Ak2	3.26	adenylate kinase 2
BE286958		3.26	predicted gene, EG668549
NM_019700	Pus1	3.26	pseudouridine synthase 1
XM_135462		3.25	predicted: *mus musculus* similar to immunoglobulin light chain variable region (LOC236047)
AB070552		3.25	*mus musculus* V102-D-J-IgG1 mRNA,
U55685		3.23	predicted gene, EG243423
NM_026444	Cs	3.23	citrate synthase
NM_177445	Dars	3.22	aspartyl-tRNA synthetase
NM_016918	Nudt5	3.21	nudix (nucleoside diphosphate linked moiety X)-type motif 5
X87228		3.21	immunoglobulin heavy chain 6 (heavy chain of IgM)
NM_028696	Obfc2a	3.21	oligonucleotide/oligosaccharide-binding fold containing 2A
NM_029573	Idh3a	3.20	isocitrate dehydrogenase 3 (NAD+) alpha
NM_019700	Pus1	3.14	pseudouridine synthase 1
BC039642	Ankrd37	3.14	ankyrin repeat domain 37
NM_146165		3.13	JTV1 gene
U39781	Igh-VJ558	3.12	immunoglobulin heavy chain (J558 family)
NM_177010	F630003A18Rik	3.12	RIKEN cDNA F630003A18 gene
NM_024282	5830417C01Rik	3.11	RIKEN cDNA 5830417C01 gene
NM_008768	Orm1	3.11	orosomucoid 1
BC043920	Frap1	3.10	FK506 binding protein 12-rapamycin associated protein 1
NM_011819	Gdf15	3.09	growth differentiation factor 15
BC060191	Cenpl	3.09	centromere protein L
NM_025462	1810009K13Rik	3.07	RIKEN cDNA 1810009K13 gene

(*Continued*)

Genbank Accession No.	Gene Symbol	Normalized Expression level (fold)	Description
NM_212451		3.06	*mus musculus* Immunoglobulin heavy chain (gamma polypeptide) (Ighg),
NM_022026	Aqp9	3.05	aquaporin 9
AF210281		3.05	immunoglobulin heavy chain 6 (heavy chain of IgM)
L22886		3.04	immunoglobulin heavy chain 6 (heavy chain of IgM)
NM_053112	Ear10	3.01	eosinophil-associated, ribonuclease A family, member 10
NM_010220	Fkbp5	3.01	FK506 binding protein 5
NM_145482	Setd4	3.01	SET domain containing 4
NM_017388	Ear3	3.00	eosinophil-associated, ribonuclease A family, member 3
NM_029662	Mfsd2	2.99	major facilitator superfamily domain containing 2
NM_007702	Cidea	2.98	cell death-inducing DNA fragmentation factor, alpha subunit-like effector A
NM_007994	Fbp2	2.95	fructose bisphosphatase 2
NM_138589	Ubfd1	2.95	ubiquitin family domain containing 1
BC038867	2810407C02Rik	2.94	RIKEN cDNA 2810407C02 gene
NM_173869		2.94	stefin A2 like 1
BC060191	Cenpl	2.93	centromere protein L
NM_025485	Mrps22	2.93	mitochondrial ribosomal protein S22
AK077046		2.92	*mus musculus* adult male testis cDNA, RIKEN full-length enriched library
NM_027764	Rcbtb1	2.92	regulator of chromosome condensation (RCC1) and BTB (POZ) domain containing protein 1
AY172876		2.91	isolate 540 immunoglobulin heavy chain variable region
BC046605	Cyp2a5	2.91	cytochrome P450, family 2, subfamily a, polypeptide 5
AK008145	Igl-V1	2.91	immunoglobulin lambda chain, variable 1
NM_008127	Gjb4	2.91	gap junction membrane channel protein beta 4
X87228		2.90	immunoglobulin heavy chain 6 (heavy chain of IgM)
NM_008637	Nudt1	2.89	nudix (nucleoside diphosphate linked moiety X)-type motif 1
NM_133245	Eraf	2.89	erythroid associated factor
NM_009921	Camp	2.88	cathelicidin antimicrobial peptide
NM_021443	Ccl8	2.88	chemokine (C-C motif) ligand 8
NM_016661	Ahcy	2.88	S-adenosylhomocysteine hydrolase
X12388		2.88	immunoglobulin heavy chain complex

(*Continued*)

Genbank Accession No.	Gene Symbol	Normalized Expression level (fold)	Description
NM_013528	Gfpt1	2.86	glutamine fructose-6-phosphate transaminase 1
NM_009474	Uox	2.86	urate oxidase
AK006122	170019H03Rik	2.84	RIKEN cDNA 1700019H03 gene
AY895789		2.83	complementarity determining region 3;
NM_029663	Eef1d	2.82	eukaryotic translation elongation factor 1 delta (guanine nucleotide exchange protein)
XM_135414	Gm189	2.82	gene model 189, (NCBI)
NM_009997	Cyp2a4	2.81	cytochrome P450, family 2, subfamily a, polypeptide 4
NM_007813	Cyp2b13	2.81	cytochrome P450, family 2, subfamily b, polypeptide 13
NM_007812	Cyp2a5	2.81	cytochrome P450, family 2, subfamily a, polypeptide 5
NM_026637	A007L17Rik	2.80	RIKEN cDNA A030007L17 gene
AK054545	Sirpb1	2.80	signal-regulatory protein beta 1
NM_028801	Muc5b	2.79	mucin 5, subtype B, tracheobronchial
NM_025300	Mrpl15	2.79	mitochondrial ribosomal protein L15
NM_031254	Trem2	2.79	triggering receptor expressed on myeloid cells 2
AK013201	Hn1l	2.78	hematological and neurological expressed 1-like
BC053478	Pctp	2.78	phosphatidylcholine transfer protein
NM_172833	Malt1	2.77	mucosa associated lymphoid tissue lymphoma translocation gene 1
NM_021525	Rcl1	2.77	RNA terminal phosphate cyclase-like 1
XM_484885	EG433326	2.76	predicted gene,
XM_132808	2510049J12Rik	2.75	RIKEN cDNA 2510049J12 gene
BF785914		2.75	DNA segment, Chr 6, Massachusetts Institute of Technology 97
BG969221		2.75	immunoglobulin kappa chain variable 28 (V28)
AB070542		2.75	*mus musculus* VH186.2-D-J-IgG1 mRNA, partial cds,
XM_487472		2.74	predicted: similar to Pyridoxal (pyridoxine, vitamin B6) kinase [*mus musculus*]
NM_026393	Nmral1	2.74	NmrA-like family domain containing 1
XM_619042		2.73	similar to Ig heavy chain V region 1B43 precursor
NM_133211	Tlr7	2.72	toll-like receptor 7
NM_198640		2.72	*mus musculus* expressed sequence AI324046 (AI324046)
U72672	Ang3	2.72	angiogenin, ribonuclease A family, member 3

(*Continued*)

Genbank Accession No.	Gene Symbol	Normalized Expression level (fold)	Description
NM_026030	Eif2s2	2.70	eukaryotic translation initiation factor 2, subunit 2 (beta)
NM_025374	Glo1	2.69	glyoxalase 1
NM_007557	Bmp7	2.69	bone morphogenetic protein 7
AB069910		2.69	*mus musculus* V303-D-J-C mu mRNA, partial cds
AK013029	Tuba1c	2.68	tubulin, alpha 1C
NM_023655	Trim29	2.66	tripartite motif protein 29
BF579422		2.65	immunoglobulin kappa chain variable 14–111
NM_025380	Eef1e1	2.65	eukaryotic translation elongation factor 1 epsilon 1
NM_153794	4933403F05Rik	2.64	RIKEN cDNA 4933403F05 gene
NM_009681	Ap3s1	2.63	adaptor-related protein complex 3, sigma 1 subunit
NM_172451	Galnt6	2.63	UDP-N-acetyl-alpha-D-galactosamine:polypeptide N-acetylgalactosaminyltransferase 6
NM_170727	Scgb3a1	2.62	secretoglobin, family 3A, member 1
NM_009997	Cyp2a4	2.62	cytochrome P450, family 2, subfamily a, polypeptide 4
NM_011082	Pigr	2.61	polymeric immunoglobulin receptor
NM_009853	Cd68	2.61	CD68 antigen
NM_016895	Ak2	2.61	adenylate kinase 2
NM_013564	Insl3	2.60	insulin-like 3
NM_001001332	BC117090	2.60	cDNA sequence BC1179090
NM_024273	4930455C21Rik	2.60	RIKEN cDNA 4930455C21 gene
NM_007557	Bmp7	2.59	bone morphogenetic protein 7
NM_172745	Tufm	2.59	Tu translation elongation factor, mitochondrial
NM_025531	Slmo2	2.59	slowmo homolog 2 (Drosophila)
NM_011399	Slc25a17	2.59	solute carrier family 25 (mitochondrial carrier, peroxisomal membrane protein), member 17
AK050325		2.58	unnamed protein product; *mus musculus* adult male liver tumor cDNA, RIKEN full-length enriched library
NM_001001332	BC117090	2.58	cDNA sequence BC1179090
NM_172552	Tdg	2.57	thymine DNA glycosylase
XM_131300	2310030N02Rik	2.57	RIKEN cDNA 2310030N02 gene
NM_175833	Cdv3	2.57	carnitine deficiency-associated gene expressed in ventricle 3
NM_011016	Orm2	2.57	orosomucoid 2
NM_025844	Chordc1	2.56	cysteine and histidine-rich domain (CHORD)-containing, zinc-binding protein 1

(*Continued*)

Genbank Accession No.	Gene Symbol	Normalized Expression level (fold)	Description
NM_146091	5730596K20Rik	2.56	RIKEN cDNA 5730596K20 gene
NM_025379	Cox7b	2.56	cytochrome c oxidase subunit VIIb
NM_011093	Pira6	2.55	paired-Ig-like receptor A6
NM_145603	Ces2	2.55	carboxylesterase 2
NM_008189	Guca1a	2.55	guanylate cyclase activator 1a (retina)
Z95476		2.55	immunoglobulin heavy chain 6 (heavy chain of IgM)
NM_008611	Mmp8	2.54	matrix metallopeptidase 8
NM_010481	Hspa9	2.54	heat shock protein 9
XM_138299		2.53	predicted: *mus musculus* similar to Igh-VJ558 protein (LOC544899)
BC057864	Cd300lf	2.52	CD300 antigen like family member F
NM_025646	Crls1	2.51	cardiolipin synthase 1
NM_011419	Jarid1d	2.51	jumonji, AT rich interactive domain 1D (Rbp2 like)
NM_007954	Es1	2.51	esterase 1
NM_181590	Shq1	2.50	SHQ1 homolog (S. cerevisiae)
NM_172733	Dera	2.50	2-deoxyribose-5-phosphate aldolase homolog (C. elegans)
NM_027552	Kynu	2.50	kynureninase (L-kynurenine hydrolase)
NM_011090	Pira3	2.49	paired-Ig-like receptor A3
NM_018866	Cxcl13	2.49	chemokine (C-X-C motif) ligand 13
M61952		2.48	gene model 1420, (NCBI)
NM_013847	Gcat	2.48	glycine C-acetyltransferase (2-amino-3-ketobutyrate-coenzyme A ligase)
NM_028112	Seh1l	2.48	SEH1-like
NM_198640		2.47	*mus musculus* expressed sequence AI324046
NM_022431	Ms4a6d	2.46	membrane-spanning 4-domains, subfamily A, member 6D
NM_026464	Wdr55	2.46	WD repeat domain 55
XM_358230		2.46	predicted: *mus musculus* similar to L-lactate dehydrogenase A chain
X67625	Igk-V21	2.46	immunoglobulin kappa chain variable 21 (V21)
AY182513		2.46	immunoglobulin heavy chain (J558 family)
NM_011150	Lgals3bp	2.45	lectin, galactoside-binding, soluble, 3 binding protein
NM_033648	Fxyd4	2.45	FXYD domain-containing ion transport regulator 4
NM_207264	BC052040	2.45	cDNA sequence BC052040
NM_183358	Gadd45gip1	2.44	growth arrest and DNA-damage-inducible, gamma interacting protein 1

(*Continued*)

Genbank Accession No.	Gene Symbol	Normalized Expression level (fold)	Description
NM_198937	Hn1l	2.44	hematological and neurological expressed 1-like
NM_007695	Chi3l1	2.44	chitinase 3-like 1
AK031392	6030422H21Rik	2.44	RIKEN cDNA 6030422H21 gene
XM_124884		2.44	predicted: *mus musculus* similar to Cytochrome P450, family 2, subfamily a, polypeptide 12 (LOC233005)
NM_025882	Pole4	2.43	polymerase (DNA-directed), epsilon 4 (p12 subunit)
NM_020001	Clec4n	2.43	C-type lectin domain family 4, member n
AF218659		2.43	predicted gene, EG211331
NM_175255	Sec24a	2.42	SEC24 related gene family, member A (S. cerevisiae)
AK040677		2.42	*mus musculus* adult male aorta and vein cDNA, RIKEN full-length enriched library
NM_021525	Rcl1	2.42	RNA terminal phosphate cyclase-like 1
NM_009266	Sephs2	2.40	selenophosphate synthetase 2
NM_025834	Proz	2.39	protein Z, vitamin K-dependent plasma glycoprotein
XM_355782		2.39	predicted: *mus musculus* immunoglobulin kappa light chain variable region Vk23 (LOC381783), mRNA
NM_177260	Tmem154	2.39	transmembrane protein 154
NM_016662	Mxd3	2.39	max dimerization protein 3
NM_027836	Ms4a7	2.39	membrane-spanning 4-domains, subfamily A, member 7
NM_029639	1600029D21Rik	2.38	RIKEN cDNA 1600029D21 gene
NM_177568	Plcb2	2.38	phospholipase C, beta 2
NM_145508	Dyrk3	2.38	dual-specificity tyrosine-(Y)-phosphorylation regulated kinase 3
NM_019939	Mpp6	2.38	membrane protein, palmitoylated 6
NM_175406	Atp6v0d2	2.37	ATPase, H+ transporting, lysosomal V0 subunit D2
NM_010220	Fkbp5	2.37	FK506 binding protein 5
NM_008866	Lypla1	2.36	lysophospholipase 1
NM_010819	Clec4d	2.35	C-type lectin domain family 4, member d
AY151141	Igl-V1	2.35	immunoglobulin lambda chain, variable 1
AF289178		2.35	immunoglobulin heavy chain 6
NM_009681	Ap3s1	2.35	adaptor-related protein complex 3, sigma 1 subunit
NM_025591	2010309E21Rik	2.35	RIKEN cDNA 2010309E21 gene
NM_023114	Apoc3	2.34	apolipoprotein C-III
NM_026950	Ociad2	2.33	OCIA domain containing 2
NM_009327	Tcf1	2.33	transcription factor 1
NM_025683	Rpe	2.33	ribulose-5-phosphate-3-epimerase

(*Continued*)

Genbank Accession No.	Gene Symbol	Normalized Expression level (fold)	Description
AK008145	Igl-V1	2.33	immunoglobulin lambda chain, variable 1
BC025836	Cpn2	2.33	carboxypeptidase N, polypeptide 2
NM_138589	Ubfd1	2.32	ubiquitin family domain containing 1
NM_182650	Hnrpa2b1	2.32	heterogeneous nuclear ribonucleoprotein A2/B1
NM_010872	Naip2	2.32	NLR family, apoptosis inhibitory protein 2
AK008381	Arl6ip2	2.32	ADP-ribosylation factor-like 6 interacting protein 2
AK019095	Sfi1	2.32	sfi1 homolog, spindle assembly associated (yeast)
NM_026276	Aasdhppt	2.32	aminoadipate-semialdehyde dehydrogenase-phosphopantetheinyl transferase
X87228		2.31	immunoglobulin heavy chain 6 (heavy chain of IgM)
NM_009014	Rad51l1	2.31	RAD51-like 1 (S. cerevisiae)
NM_021522	Usp14	2.31	ubiquitin specific peptidase 14
NM_031884	Abcg5	2.31	ATP-binding cassette, sub-family G (WHITE), member 5
NM_025878	Mrps18b	2.30	mitochondrial ribosomal protein S18B
NM_009705	Arg2	2.30	arginase type II
NM_007802	Ctsk	2.29	cathepsin K
NM_013799	Ate1	2.29	arginine-tRNA-protein transferase 1
NM_133686	Qprt	2.29	quinolinate phosphoribosyltransferase
NM_025589	Rpl36al	2.28	ribosomal protein L36a-like
NM_145154	Angptl6	2.28	angiopoietin-like 6
NM_009423	Traf4	2.28	Tnf receptor associated factor 4
NM_013651	Sf3a2	2.27	splicing factor 3a, subunit 2
AK011495	Lman1	2.27	lectin, mannose-binding, 1
XM_143595	EG229879	2.27	predicted gene, EG229879
NM_175833	Cdv3	2.27	carnitine deficiency-associated gene expressed in ventricle 3
NM_010391	H2-Q10	2.27	histocompatibility 2, Q region locus 10
NM_178309	Brip1	2.26	BRCA1 interacting protein C-terminal helicase 1
BC048732	Tmem103	2.26	transmembrane protein 103
XM_484186		2.26	predicted: *mus musculus* similar to anti-poly(dC) monoclonal antibody heavy chain (LOC432703), mRNA
NM_026149	Nudcd1	2.26	NudC domain containing 1
NM_023219	Slc5a4b	2.25	solute carrier family 5 (neutral amino acid transporters, system A), member 4b
NM_011090	Pira3	2.25	paired-Ig-like receptor A3

(*Continued*)

Genbank Accession No.	Gene Symbol	Normalized Expression level (fold)	Description
NM_025412	Pycrl	2.25	pyrroline-5-carboxylate reductase-like
NM_078478	Ghitm	2.25	growth hormone inducible transmembrane protein
XM_488239		2.24	*mus musculus* similar to Ig heavy chain V region VH558 A1/A4 precursor (LOC436124), mRNA
NM_212444	Gyk	2.24	glycerol kinase
NM_027346	Ccdc44	2.24	coiled-coil domain containing 44
BC025535	Fcgr1	2.23	Fc receptor, IgG, high affinity I
NM_177544	Ang4	2.23	angiogenin, ribonuclease A family, member 4
NM_025433	Rpl7l1	2.23	ribosomal protein L7-like 1
NM_028788	1300002K09Rik	2.23	RIKEN cDNA 1300002K09 gene
NM_178605	D13Wsu177e	2.23	DNA segment, Chr 13, Wayne State University 177, expressed
AK017820	Mrpl15	2.23	mitochondrial ribosomal protein L15
AK006993	1700084J12Rik	2.23	RIKEN cDNA 1700084J12 gene
NM_030597	Lsm2	2.22	LSM2 homolog, U6 small nuclear RNA associated (S. cerevisiae)
NM_146020	C730027E14Rik	2.22	RIKEN cDNA C730027E14 gene
NM_144869	BC021614	2.22	cDNA sequence BC021614
NM_007856	Dhcr7	2.22	7-dehydrocholesterol reductase
NM_178643	C230052I12Rik	2.22	RIKEN cDNA C230052I12 gene
NM_030250	D10Ertd438e	2.21	DNA segment, Chr 10, ERATO Doi 438, expressed
NM_027250	2010305A19Rik	2.21	RIKEN cDNA 2010305A19 gene
NM_020010	Cyp51	2.21	cytochrome P450, family 51
NM_029742	2410127E18Rik	2.20	RIKEN cDNA 2410127E18 gene
NM_013463	Gla	2.19	galactosidase, alpha
NM_022316	Smoc1	2.19	SPARC related modular calcium binding 1
NM_010481	Hspa9	2.19	heat shock protein 9
XM_355832	Gm1077	2.19	gene model 1077, (NCBI)
NM_021522	Usp14	2.18	ubiquitin specific peptidase 14
NM_008194	Gyk	2.17	glycerol kinase
NM_026835	Ms4a6d	2.15	membrane-spanning 4-domains, subfamily A, member 6D
NM_012055	Asns	2.15	asparagine synthetase
NM_175380	Gpd1l	2.14	glycerol-3-phosphate dehydrogenase 1-like
NM_019633		2.14	*mus musculus* recombinant antineuraminidase single chain Ig VH and VL domains (LOC56304), mRNA
NM_011338	Ccl9	2.14	chemokine (C-C motif) ligand 9

(*Continued*)

Genbank Accession No.	Gene Symbol	Normalized Expression level (fold)	Description
NM_021281	Ctss	2.14	cathepsin S
NM_008194	Gyk	2.14	glycerol kinase
NM_024282	5830417C01Rik	2.14	RIKEN cDNA 5830417C01 gene
AK007467	1810012P15Rik	2.14	RIKEN cDNA 1810012P15 gene
NM_153776	Tmem121	2.14	transmembrane protein 121
NM_134158	Cd300d	2.13	Cd300D antigen
NM_025606	Mrpl16	2.13	mitochondrial ribosomal protein L16
NM_010130	Emr1	2.13	EGF-like module containing, mucin-like, hormone receptor-like sequence 1
NM_007757	Cpox	2.13	coproporphyrinogen oxidase
AK007931	Ela1	2.13	elastase 1, pancreatic
NM_184052	Igf1	2.12	insulin-like growth factor 1
BC080787		2.12	immunoglobulin kappa chain variable 28 (V28)
NM_029796	Lrg1	2.11	leucine-rich alpha-2-glycoprotein 1
NM_011081	Piga	2.11	phosphatidylinositol glycan anchor biosynthesis, class A
NM_007861	Dld	2.11	dihydrolipoamide dehydrogenase
NM_007883	Dsg2	2.11	desmoglein 2
NM_016960	Ccl20	2.11	chemokine (C-C motif) ligand 20
NM_008255	Hmgcr	2.09	3-hydroxy-3-methylglutaryl-Coenzyme A reductase
NM_008176	Cxcl1	2.09	chemokine (C-X-C motif) ligand 1
NM_027187	Rnaseh2a	2.09	ribonuclease H2, large subunit
NM_019573	Wwox	2.08	WW domain-containing oxidoreductase
NM_011610	Tnfrsf1b	2.08	tumor necrosis factor receptor superfamily, member 1b
NM_133862	Fgg	2.08	fibrinogen, gamma polypeptide
AK002912	Pycrl	2.08	pyrroline-5-carboxylate reductase-like
NM_030611	Akr1c6	2.08	aldo-keto reductase family 1, member C6
NM_133819	Ppp1r15b	2.08	protein phosphatase 1, regulatory (inhibitor) subunit 15b
BG968852		2.08	gene model 1499, (NCBI)
NM_019976	Psrc1	2.08	proline/serine-rich coiled-coil 1
NM_172514	Tmem71	2.07	transmembrane protein 71
NM_020275	Tnfrsf10b	2.07	tumor necrosis factor receptor superfamily, member 10b
NM_020010	Cyp51	2.07	cytochrome P450, family 51
NM_030197	2700078E11Rik	2.07	RIKEN cDNA 2700078E11 gene

(*Continued*)

Genbank Accession No.	Gene Symbol	Normalized Expression level (fold)	Description
AK039267	Aldoc	2.07	aldolase 3, C isoform
NM_010579	Itgb4bp	2.06	integrin beta 4 binding protein
M94350	Igl-V1	2.06	immunoglobulin lambda chain, variable 1
NM_008813	Enpp1	2.05	ectonucleotide pyrophosphatase/phosphodiesterase 1
NM_025509	2310008M10Rik	2.05	RIKEN cDNA 2310008M10 gene
XM_284750		2.05	6-phosphofructo-2-kinase/fructose-2, 6-biphosphatase 1
AK018541	9030607L17Rik	2.04	RIKEN cDNA 9030607L17 gene
BC066035	Zranb3	2.04	zinc finger, RAN-binding domain containing 3
XM_127250		2.04	E2F transcription factor 3.
NM_027250	2010305A19Rik	2.04	RIKEN cDNA 2010305A19 gene
XM_355437		2.03	SIRP beta 1 like 1 protein
NM_010188	Fcgr3	2.03	Fc receptor, IgG, low affinity III
NM_026165	Slc25a46	2.02	solute carrier family 25, member 46
NM_026630	2410116G06Rik	2.02	RIKEN cDNA 2410116G06 gene
NM_010249	Gabpb1	2.01	GA repeat binding protein, beta 1
NM_133752	Opa1	2.01	optic atrophy 1 homolog (human)
NM_016792	Txnl1	2.01	thioredoxin-like 1
X96776		2.00	C2-Fcgamma fusion protein
